# Targeting a Moving Threshold: Antigen Density as a Design Constraint for Particulate Drug Delivery in HER2-Low and HER2-Ultralow Breast Cancer—A Narrative Review

**DOI:** 10.3390/pharmaceutics18101218

**Published:** 2026-09-25

**Authors:** Vasile Adrian Sescu, Alexandru Filipescu, Iustina Petra Solomon Condriuc, Mihaela Burac, Ioana Sadiye Scripcariu, Demetra Socolov, Răzvan Socolov, Alexandru Cărăuleanu

**Affiliations:** 1 Grigore T. Popa University of Medicine and Pharmacy, 700115 Iasi, Romaniaioana.scripcariu@umfiasi.ro (I.S.S.); demetra.socolov@umfiasi.ro (D.S.); razvan.socolov@umfiasi.ro (R.S.); ale.carauleanu@umfiasi.ro (A.C.); 2 Department of Obstetrics and Gynecology, Cuza Voda Hospital, 700038 Iasi, Romania; 3 Department of Obstetrics and Gynecology, “Carol Davila” University of Medicine and Pharmacy, 050474 Bucharest, Romania; 4 Department of Obstetrics and Gynecology, Elias University Emergency Hospital, 050474 Bucharest, Romania; 5 Department of Obstetrics, Gynecology and Human Reproduction, “Nicolae Testemițanu” State University of Medicine and Pharmacy, MD-2004 Chișinău, Moldova; 6 Department of Obstetrics and Gynecology, Clinical Hospital of Obstetrics and Gynecology “Elena Doamna”, 700038 Iasi, Romania

**Keywords:** HER2-low breast cancer, HER2-ultralow, antigen density, receptor density, particulate drug delivery, nanoparticle, active targeting, multivalency, avidity, nanomedicine

## Abstract

Trastuzumab deruxtecan has made breast tumours with minimal HER2 expression therapeutically actionable, and the descriptors HER2-low and HER2-ultralow now carry regulatory weight. Both were defined by immunohistochemical convention and trial eligibility rather than by calibrated measurement. For a receptor-directed particulate carrier the operative question is not whether HER2 is detectable, but whether enough surface-accessible receptor is present, and suitably distributed, to sustain multivalent attachment, clustering, internalisation and payload transfer. This narrative review asks whether principles established in HER2-overexpressing models, in non-HER2 receptor systems and on synthetic interfaces can defensibly be extrapolated to the HER2-low setting, keeping those evidence classes distinct. Antigen density acts at several stages of the delivery cascade rather than at binding alone, jointly with carrier design and tumour transport. An analytical constraint compounds this: fixed-tissue methods report tissue HER2 abundance but do not selectively quantify the accessible receptor pool a carrier can engage. Within the literature identified, no engagement threshold generalisable across platforms was found; the single measured value belongs to one liposomal system studied in vitro. Lesion-level carrier deposition varied widely in both human imaging datasets identified, which illustrate transport heterogeneity but are not comparable. Targetability is platform-specific, and should be measured rather than inferred from categorical HER2 status. No validated model links receptor abundance, ligand density, avidity and internalisation to biological response, so the framework offered is operational.

## 1. Introduction

Classification of human epidermal growth factor receptor 2 (HER2) status in breast cancer was built for a single decision. Immunohistochemistry (IHC) and in situ hybridisation were developed to identify the minority of tumours whose ERBB2 amplification made them candidates for trastuzumab, with scoring criteria optimised to separate overexpression from everything else [1]. The assay performs that task well, but was not designed to resolve differences among the tumours it classifies as negative.

That assumption became consequential when trastuzumab deruxtecan (T-DXd) produced clinically meaningful activity in tumours scored IHC 1+ or IHC 2+ without gene amplification. In DESTINY-Breast04, in patients selected as HER2-low by central testing, T-DXd extended median progression-free survival from 5.1 to 9.9 months (hazard ratio [HR] 0.50; 95% confidence interval [CI] 0.40–0.63) and median overall survival from 16.8 to 23.4 months (HR 0.64; 95% CI 0.49–0.84) [2]. The survival advantage held on extended follow-up [3]. DESTINY-Breast06 then enrolled, alongside a HER2-low population, an exploratory HER2-ultralow cohort [4]. Approvals followed for HER2-low in 2022 and for HER2-low or HER2-ultralow hormone receptor-positive disease in 2025, the latter tied to a single named antibody clone as companion diagnostic [5,6].

A clinical vocabulary therefore exists for tumours expressing HER2 at levels the assay was not built to quantify, but it does not supply the variable a targeted delivery system requires. A particulate carrier meets a cell surface presenting some number of accessible receptors per unit membrane area, in some spatial pattern, embedded in tissue of some permeability. Whether binding is stable, whether it proceeds to clustering and internalisation, and whether internalisation delivers enough payload to matter are functions of that physical situation, not of the label attached to it.

“Particulate drug delivery” is used here for colloidal carriers that transport a payload as a discrete particle and engage the cell surface through multiple ligands at once: liposomes and immunoliposomes, lipid nanoparticles, polymeric nanoparticles and micelles, dendrimers, protein cages, inorganic and hybrid constructs, and engineered extracellular vesicles. No fixed size cut-off was imposed for this review, given the heterogeneous dimensions of the platforms considered; classification instead rested on colloidal transport behaviour and multivalent surface engagement. Antibody–drug conjugates (ADCs) fall outside that definition and are treated as mechanistic comparators. The distinction is physical: an antibody is bivalent, roughly 10 nm across and circulates for weeks, whereas a liposome or polymeric nanoparticle is one to two orders of magnitude larger, carries tens to hundreds of ligands, extravasates differently, and is cleared predominantly by the mononuclear phagocyte system. Clinical success of a HER2-directed ADC in HER2-low disease therefore does not license the inference that a HER2-targeted nanoparticle will behave similarly.

How much accessible HER2, distributed in what way, does a particulate drug-delivery system require in order to achieve therapeutically meaningful targeting? The analysis is mechanistic and translational rather than confirmatory, because almost no primary evidence has been generated in HER2-low or HER2-ultralow tumours: what exists comes from HER2-overexpressing models, non-HER2 receptor systems, antibody and conjugate studies, and synthetic interfaces. The question is therefore whether principles derived from those sources can defensibly be extrapolated downward in receptor abundance, and where that extrapolation breaks down; no claim is made that the phenomena discussed have been demonstrated in clinical disease. The title proposes antigen density as a design constraint, and the argument is built to test that proposition rather than assume it. It would be weakened by delivery failing well above any measured engagement threshold, or by outcome remaining unchanged when receptor availability is raised without altering transport.

The question falls between two bodies of literature. Reviews of HER2-targeted nanoparticles catalogue platforms, ligand formats and conjugation chemistries, treating HER2 positivity as an entry condition rather than a graded variable [7], whereas reviews and consensus statements on HER2-low disease address pathology and ADC therapy without reference to particulate carriers. Neither asks what quantity of receptor a multivalent carrier needs. What follows is organised around that quantity, separating receptor abundance, surface density, surface-accessible receptor and fixed-tissue measurement, which the delivery literature routinely conflates.

Approach to the literature. PubMed/MEDLINE, Scopus and Web of Science were consulted through 25 August 2026 across four domains: HER2 expression and its classification in breast cancer; receptor or antigen density as a determinant of targeted delivery; particulate delivery platforms; and the physical chemistry of multivalent binding, including avidity, ligand density, internalisation and intratumoural transport. English-language literature was considered. ADCs were treated separately, as mechanistic comparators. Primary research was preferred, with guidelines, consensus documents and regulatory approval notices used for definitions and regulatory status, and registry entries where no peer-reviewed report exists. Reference lists of retrieved articles were screened for further primary sources, and recent work was prioritised while retaining earlier studies that remain the original source of a mechanistic principle. Sources were selected purposively for their relevance to the review question and to the mechanistic synthesis rather than to provide an exhaustive inventory, and reporting follows the principles of the Scale for the Assessment of Narrative Review Articles (SANRA) [8]. Statements about what was or was not found describe this body of literature, not absence of evidence. For the present revision, the search was extended to 19 September 2026 and broadened to four further domains: receptor trafficking and the nanoscale organisation of HER2 at the cell membrane; the analytical performance and comparability of protein-quantification assays; single-cell and spatially resolved profiling of tumour tissue; and computational and mathematical models of receptor-mediated uptake and of nanoparticle transport. Regulatory guidance documents were consulted directly on the issuing agency’s website.

## 2. HER2-Low and HER2-Ultralow: Clinical Categories Versus Quantitative Targets

### 2.1. Evolution of HER2 Classification

The operative scoring criteria remain those of the 2018 ASCO/College of American Pathologists (CAP) focused update, reaffirmed in 2023 [1,9]. They define a four-tier IHC scale intended to identify HER2-positive disease and triage equivocal cases to in situ hybridisation. The lower two tiers were not constructed to be informative in themselves, and no calibration converts them into receptor abundance.

### 2.2. The Current Meaning of HER2-Low

HER2-low denotes an IHC score of 1+, or 2+ with negative in situ hybridisation (ISH). Four labels must be kept apart. HER2-negative covers everything that is not HER2-positive and therefore includes HER2-low; HER2-zero denotes IHC 0 as historically reported, which current recommendations subdivide into HER2-ultralow and HER2-null (Section 2.3), so studies predating that subdivision cannot be resolved into the newer categories. The category is common: central testing in four prospective neoadjuvant trials classified 47.5% of HER2-non-amplified tumours as HER2-low [10] and targeted sequencing of metastatic disease gave 47% [11], with prevalence differing sharply by hormone receptor status (65.4% versus 36.6% in triple-negative disease) [12]. Reported figures span roughly 45% to three-fifths of HER2-negative tumours depending on denominator, era and laboratory [13,14].

Whether HER2-low is a biological entity is a separate question from whether it is a useful treatment category, and the evidence does not settle it in the affirmative. Pooled neoadjuvant data showed higher three-year disease-free survival in HER2-low than HER2-zero tumours (83.4% versus 76.1%, *p* = 0.0084) [10], but the difference was confined to hormone receptor-negative disease and is confounded by subtype, HER2-low tumours being 64.0% hormone receptor-positive against 36.7% of HER2-zero. Meta-analysis of 42 studies and approximately 1.8 million patients found prognostic effects of marginal magnitude [15]; genomic characterisation of 1039 metastatic tumours found no alteration other than ERBB2 copy number surviving correction for multiple testing [11]; and transcriptomic profiling in 1320 of a 3689-patient HER2-negative cohort found no differentially expressed genes between HER2-low and HER2-zero triple-negative disease [12]. Studies reporting distinct features are smaller or show heterogeneity within the category itself [16,17]. One genuinely distinct subgroup exists, defined by 17q12 deletion with heterozygous ERBB2 loss, but it sits at the bottom of the expression range rather than at the HER2-low/HER2-zero boundary [18].

### 2.3. The Current Meaning of HER2-Ultralow

HER2-ultralow is the more recently operationalised of the two descriptors, and the less independently validated as a subgroup. International expert consensus defines it as faint or barely perceptible, incomplete membrane staining in more than 0% and up to 10% of tumour cells, with complete absence designated HER2-null [19]. The definition originated in clinical trials rather than diagnostic practice, at a boundary already known to be the least analytically reproducible point on the scale (Section 2.4).

The clinical evidence base is correspondingly narrow. In DESTINY-Breast06, 153 of 866 randomised patients had HER2-ultralow disease, and that population was explicitly exploratory; the primary endpoint was progression-free survival in the HER2-low population, extended from 8.1 to 13.2 months (HR 0.62; 95% CI 0.52–0.75) [4]. Two non-identical sets of ultralow figures are in circulation and should be cited separately: 13.2 versus 8.3 months with an HR of 0.78 (95% CI 0.50–1.21) in the trial publication [4], and 15.1 versus 8.3 months with an HR of 0.76 (95% CI 0.49–1.17) in the regulatory review of the same subgroup [6]. Two things follow and should not be conflated. The category has established regulatory and clinical relevance, with approval resting on consistency of effect within a trial that met its primary endpoint elsewhere; the subgroup estimate itself is exploratory, not independently powered, and bounded by confidence intervals crossing unity. Clinical utility in this population is supported, but a precise magnitude of benefit within it is not yet established.

The 2023 ASCO/CAP guideline update named both categories and considered the creation of new HER2 result categories premature at that time, while accepting that distinguishing IHC 0 from 1+ had become clinically relevant because of trial entry criteria [9]. That formal guideline has not been superseded, but the position around it has moved. Subsequent CAP breast biomarker reporting templates have incorporated trial-based reporting language, subdividing the IHC 0 category and offering standardised optional comments identifying the HER2-low and HER2-ultralow definitions used in DESTINY-Breast04 and DESTINY-Breast06 [20]; the 2025 regulatory decision adopted the terms; and the 2026 international consensus provides reporting definitions for both categories and for HER2-null [19]. The European consensus process had earlier reached a parallel conclusion by a different route, treating HER2-low as a therapeutic rather than a biological category [21]. These categories are best understood as regulatory and operational constructs of demonstrated clinical utility, not as measurements of receptor abundance.

### 2.4. Analytical Uncertainty at Low HER2 Expression

Reproducibility at the bottom of the scale is poor, consistently across designs. When 18 pathologists scored scanned breast biopsies, concordance was 26% for the 0 versus 1+ distinction against 58% for 2+ versus 3+, and CAP proficiency surveys covering 1391 to 1452 laboratories showed 19% of cases generating 70% concordance or less for 0 versus 1+ [22]. A ring study of 77 pathologists across 14 countries found positive percentage agreement for HER2-0 of 74.6%, rising to 89.2% after four hours of structured training [23], and a 45-laboratory comparison gave 68.9% inter-laboratory concordance overall (κ = 0.566), falling to 57.6% for slides scored IHC 1+ [24]. Agreement is highest for 3+ and for unequivocally unstained tumours, and lowest at the 0/1+ boundary on which the HER2-low category depends.

Pre-analytical and reagent variables move cases across that boundary. Cold ischaemia reduced HER2 staining in 48% of non-refrigerated specimens against 24% of refrigerated specimens [25], and decalcification potentially influenced treatment decisions in 2 of 23 patients [26]. Assay choice alone changed category assignment, the proportion classified HER2-low being 35% with one validated assay against 19% with another in the same 119 tumours (*p* < 0.01) [27], with antibody clone shifting the distribution of 0 and 1+ scores in an independent series [24]. Sampling adds a further layer: among 1387 paired core biopsy and resection specimens, concordance was 99.0% for the binary negative/positive call but 78.5% (κ = 0.587) for a zero/low/positive classification, with 42.8% of core-biopsy HER2-zero cases reclassified as HER2-low on resection [28]—an asymmetry reproduced in 5610 consecutive patients, where 49.7% converted at excision [29].

These sources of variability are not independent, and their combination is what determines category assignment at the bottom of the scale. Reader-level disagreement, laboratory-level assay configuration, antibody clone, pre-analytical handling and the specimen submitted each move a proportion of cases across the 0 versus 1+ boundary, so the same tumour can be reported differently by two competent laboratories without either being in error. The underlying reason is that the scoring criteria were developed and validated for the HER2-positive versus HER2-negative decision rather than for discrimination within the negative range [1,9]; no reference material, calibrator or proficiency standard currently anchors the lower boundary, and the analytical characteristics of the assays that might do so are compared in Section 10.3. The consequence for the present argument is specific rather than general: the variance in the category label is largest precisely where the receptor abundance of interest is smallest, so a categorical result carries less information about the surface a carrier will meet in HER2-low and HER2-ultralow disease than it does in HER2-positive disease.

### 2.5. Why IHC Categories Cannot Be Converted into Receptor Number

Chromogenic IHC is an ordinal, semi-quantitative assay read by eye and optimised for a distinction between two categories above the one now asked of it. Calibrated quantitative immunofluorescence traceable to mass spectrometry placed HER2 in unamplified cell lines at 2–20 attomol/mm^2^, with 67% of 364 tumours above the limit of quantification yet below amplified levels [30], and a high-sensitivity version applied to 316 core biopsies found 71% of pathologist-scored IHC 0 cases above its limit [31]. Targeted mass spectrometry in gastro-oesophageal tumours showed a greater than hundredfold spread within the IHC 3+/amplified group alone [32]; the tissue type differs, the point about within-category dispersion does not.

Absolute receptor numbers, where measured, are method-dependent in ways that matter for any attempt to attach numbers to categories. Bead-calibrated flow cytometry on unpermeabilised cells placed the HER2-positive lines SK-OV-3, BT-474, NCI-N87 and SK-BR-3 between 1.42 × 10^6^ and 2.03 × 10^6^ surface HER2 molecules per cell, and the weakly expressing and negative lines MDA-MB-231, MCF-7, OVCAR3 and MDA-MB-453 between 2.81 × 10^4^ and 2.75 × 10^5^ [33]. A second bead-calibrated determination gave 8203 receptors per cell for T-47D, 47,050 for MDA-MB-453, 277,096 for BT-474 and 456,584 for AU-565, and the calibrated quantitative immunohistochemistry built on those calibrators reported 10,375–178,649 receptors per cell in tumour tissue, discordant with categorical scoring [34]. Two lines were measured by both—BT-474 at 1.82 × 10^6^ and 277,096, MDA-MB-453 at 2.75 × 10^5^ and 47,050—each differing by close to sixfold. Absolute receptor abundance is therefore assay-dependent, and reported values should be cited with the method attached rather than pooled onto a common scale.

A further limitation applies to all of these assays and is rarely stated. Calibrated immunohistochemistry, quantitative immunofluorescence and targeted mass spectrometry are performed on fixed tissue and report tissue HER2 abundance or signal; they do not selectively quantify the live, extracellularly accessible plasma-membrane pool an incoming carrier can engage, which is smaller, dynamic and sustained by endosomal recycling (Section 3.4). Bead-calibrated flow cytometry on unpermeabilised cells does report surface-available receptor [33], but is not applicable to archival tissue. We identified no clinically validated tissue assay that selectively quantifies the surface-accessible HER2 pool relevant to carrier engagement, and the gap between tissue-level and accessible receptor is unquantified in human tumours.

How much of the HER2 in a tumour cell is available to an extracellular binder is itself measurable in principle, and where it has been measured, the two quantities have differed. In the trastuzumab-resistant line JIMT-1, the mean number of trastuzumab binding sites per cell was approximately one fifth of the number of erbB2 molecules expressed, with 5–10% of cells binding roughly tenfold more antibody than the main population; trastuzumab Fab fragments and an antibody directed at a membrane-distal epitope bound more efficiently than intact trastuzumab, and knockdown of the membrane mucin MUC4 increased trastuzumab binding [35]. That is one resistant line with a specific masking mechanism, and no general correction factor follows from it. What it establishes is narrower and sufficient for the present purpose: expressed receptor and binder-accessible receptor are separable quantities that can differ several-fold in the same cell, and it is the second that a multivalent carrier engages.

No defensible conversion exists between an IHC category and a receptor-per-cell interval: HER2-low does not correspond to a number, and HER2-ultralow does not correspond to a reproducible quantitative interval. Because measured abundance overlaps substantially across adjacent categories, two tumours reported identically may present materially different surfaces to a carrier. Table 1 sets out what each category does, and does not, specify.

## 3. Antigen Density as an Engineering Variable

Antigen density enters particulate delivery not as a single parameter but as a term recurring at several independent steps. Four quantities are distinguished throughout: receptor abundance, receptors per cell; surface density, receptors per unit membrane area; surface-accessible receptor, the fraction available at the plasma membrane for engagement; and fixed-tissue HER2 measurement. Almost all published values are receptor abundance, and where cell-surface area was not reported, receptor number per cell is treated as an operational measure of abundance rather than a true surface density; the two are not interchangeable.

A second distinction runs through the review and is applied explicitly from here on, because the evidence differs in how directly it bears on HER2-low particulate delivery. Five classes are separated so that mechanistic analogy is never given the inferential weight of direct evidence. Level A: a HER2-targeted particulate system in which HER2 abundance was quantified. Level B: the same without calibrated receptor quantification. Level C: a HER2-targeted antibody or conjugate informing binding, penetration, trafficking or payload behaviour. Level D: a particulate system targeting a receptor other than HER2. Level E: a mechanistic analogue—synthetic surfaces, model membranes, nucleic-acid scaffolds, and theoretical or computational work. These label the type of source, not study quality, certainty or risk of bias. Two kinds of sources fall outside the scheme and are labelled as such wherever they appear: untargeted carriers used to manipulate HER2 expression, and general particulate evidence on transport, clearance and corona behaviour involving no receptor targeting. Level A evidence is scarce, and much of what follows is Level C to E; where that is so, it is stated. Evidence levels are stated wherever a mechanistic principle derived from one class is applied to another, and every entry in the summary tables below carries one.

### 3.1. Receptor Abundance and the Probability of Productive Engagement

A soluble antibody binds through one or two paratopes, and its retention is governed by monovalent affinity; a particle binds through many ligands at once, and its retention is governed by the collective off-rate of an ensemble of weak contacts. Under appropriate physicochemical conditions, multivalent particle binding can therefore depend on receptor density in a strongly non-linear, superselective manner rather than proportionally, depending on valency, per-bond affinity, ligand mobility and the steric environment of the particle surface.

Theoretical treatment of this behaviour, termed superselectivity, showed that the number of distinct ligand–receptor binding arrangements available to a multivalent object grows highly non-linearly with receptor surface coverage, so a modest increase in density can produce a disproportionate increase in bound fraction, most strongly when individual bonds are weak [40]. The prediction has been confirmed on defined surfaces [43]; the transition proved tunable through probe design [44] and has been resolved directly by light microscopy with Förster resonance energy transfer [45]. At fluid interfaces, multivalent probes retain superselectivity, and receptor clustering enhances it and shifts it to lower densities [46,47].

The corollary matters at the low end: where such a regime operates, an architecture sharply selective above a given density may show threshold-like or steeply non-linear loss of engagement below it rather than a gradual decline. This work is Level E—defined synthetic surfaces, colloidal probes and model membranes—a plausible mechanistic analogue for carrier binding to a tumour-cell membrane that has not been demonstrated in HER2-low tumour tissue, and should be read as prediction rather than an observed property of any clinical carrier.

### 3.2. Affinity Versus Avidity

Increasing per-ligand affinity is an intuitive response to scarce antigen, but may compromise intratumoural distribution. In single-chain variable fragment panels spanning 10^−7^ to 10^−11^ M against the same epitope, tumour retention did not improve beyond approximately 10^−9^ M, and the highest-affinity variants produced perivascular rather than diffuse staining [48]. Across variants differing 500-fold in KD, the highest-affinity construct remained within 20.4 ± 7.5 µm of the vasculature while the lowest reached 84.8 ± 12.8 µm, with intermediate affinity giving the greatest overall accumulation [49]. High affinity favours binding to the first receptors encountered, restricting deeper distribution.

For particles, per-ligand affinity is only one input into avidity, and the relationship is architecture-dependent. Free-energy computation of nanocarriers displaying antibody at varying surface coverage predicted a coverage below which apparent affinity collapses, validated experimentally against endothelial ICAM-1 [50]; particles bearing a bimodal polymer brush were selected for receptor density through a steric barrier surmountable only by many simultaneous bonds [51].

### 3.3. Ligand Density on the Carrier Surface

Ligand density is the parameter most often adjusted and least often measured. Where it has been measured, three findings recur: the optimum is intermediate, nominal and functional density diverge, and excess carries costs beyond waste.

On the first point, HER2 affibody–conjugated iron oxide particles compared across ligand density, particle size and target receptor level showed an intermediate optimum holding across all three [52]. In peptide-targeted liposomes, uptake plateaued at roughly 2% peptide density, and a conventionally constructed conjugate gave no advantage over the untargeted particle [53], while a companion study varying linker length and ligand hydrophilicity produced 27- to 80-fold uptake gains at fixed peptide density [54]. With trastuzumab counted directly at 330 ± 30, 480 ± 110 and 690 ± 80 antibodies per liposome, spheroid viability did not differ significantly across that twofold range, although all targeted formulations beat the plain liposome [55].

Nominal and functional density diverge sharply. Single-molecule counting showed only a minority of conjugated fragments remaining accessible, so a twentyfold increase in conjugated antibody yielded roughly a twofold increase in functional binding sites [56]; reported ligand densities are therefore systematically higher than those that participate in binding.

Excess ligand also carries costs beyond waste. In a lipid nanoparticle system with single-particle quantification of functional ligand, targeting CD8 on T cells rather than a tumour antigen, in vivo performance peaked at low ligand density and higher densities degraded the target receptor [57]. In one of the few studies controlling ligand number exactly, gold particles bearing precisely one or two trastuzumab molecules behaved oppositely: two were superior in vitro, one gave approximately twofold greater tumour accumulation in vivo [58].

### 3.4. Receptor Clustering, Mobility, Trafficking and Internalisation

Binding and internalisation are separable events with different requirements. Receptor lateral mobility governs whether bound ligands can recruit further receptors into a contact zone, and on fluid membranes small inter-receptor attractions shift the binding transition to lower densities [46,47]. Spatial arrangement matters independently of number, on Level E evidence from non-HER2 systems: halving inter-ligand spacing on DNA origami from 17.5 nm to 7 nm approximately doubled macrophage engulfment on contact [59]; hexagonal Fas-ligand arrays at 10 nm spacing raised signalling efficiency by two orders of magnitude over soluble ligand [60]; and valency titration of an insulin-receptor construct was non-monotonic [61]. Whether the same rules govern HER2 engagement by a soft, polydisperse carrier is untested.

Direct measurement of HER2 organisation on breast cancer membranes supports the relevance of that geometry to HER2 itself, while showing how far it is from uniform. Single-molecule localisation microscopy with fluorophore-labelled trastuzumab, which counts only receptor retaining the trastuzumab-binding extracellular domain, gave detected surface densities of 119, 77 and four molecules per µm^2^ for BT-474, SK-BR-3 and MDA-MB-468 respectively, with 49% and 59% of receptor in clusters of more than two molecules in the two HER2-positive lines, mean cluster radii of 21 and 24 nm, and no clusters detectable in the low-expressing line [62]. Earlier localisation microscopy of Her2/neu on plasma-membrane sections reported 20,637 clusters of 67 nm mean diameter across 176 cells from two mammary carcinoma lines and a healthy donor, with clustering statistics that distinguished the three cell types [63], and correlative fluorescence and liquid-phase electron microscopy of SK-BR-3 cells at 3 nm resolution found homodimers and higher-order clusters concentrated in membrane ruffles while topographically flat regions showed HER2 distributed at random [64]. Two points follow for a multivalent carrier. These are among the few HER2 measurements expressed per unit membrane area rather than per cell, and they show that the two are not interchangeable; local density within a cluster exceeds the cell mean, so the density a carrier footprint samples depends on where it lands. Whether particulate binding exploits that is untested for HER2, although receptor clustering sharpens multivalent selectivity and shifts it to lower mean densities on model membranes (Level E) [46,47]. The two scales should not be reconciled by arithmetic: converting receptors per cell into receptors per µm^2^ requires a value for plasma membrane area that neither source reports, and the implied area is large enough to indicate that the two methods do not count the same population—localisation microscopy counting probe-accessible molecules detected within the imaged membrane region, calibrated flow cytometry counting antibody-binding sites over the whole cell surface [33,34,62]. That is the point rather than an inconsistency: they are different measurements, and neither converts into the other.

HER2 is a demanding target for internalisation-dependent strategies. Its surface pool is dynamic and sustained by endosomal recycling, with trastuzumab recycling passively rather than redirecting that traffic [65], and modelled internalisation rates differ by around three orders of magnitude between morphologically distinct cell states [66]. In HER2-overexpressing lines, internalisation, degradation and efflux half-times of 6–14, 18–25 and 44–73 h were measured, with internalisation and efflux governing intracellular payload per cell [67]. Receptor abundance sets the ceiling on uptake; trafficking determines how much is realised.

Two features of that traffic bear directly on what a circulating carrier encounters. The first is that the accessible pool is a steady state rather than a stock. Quantitative immunoelectron microscopy and surface-quenching kinetics in breast carcinoma cells showed surface ErbB2 to be maintained by constitutive endocytosis balanced against efficient recycling, with trastuzumab recycling passively alongside it and geldanamycin lowering surface receptor by redirecting endosomal sorting toward degradation rather than by accelerating uptake [65]. A contemporaneous account holds instead that ErbB2 associates preferentially with membrane protrusions and is largely excluded from clathrin-coated pits, becoming internalisation-competent only once efficiently cross-linked [68]; the two models have not been reconciled, and reviews of ErbB2 trafficking continue to treat its resistance to down-regulation as unsettled [69]. Both nonetheless converge on the same operational point: surface HER2 is continuously replenished, and the fraction of total cellular HER2 exposed at the plasma membrane at any instant is neither fixed nor equal to the total.

The second feature is that trafficking behaviour is itself a function of expression level. Across a panel of breast and prostate lines spanning a wide HER2 range, high-expressing cells recycled HER2 efficiently, lost little receptor on antibody treatment and retained antibody at the plasma membrane, whereas low-expressing cells cleared antibody rapidly from the surface, lost substantially more receptor and showed no detectable recycling; expression level rather than tissue of origin predicted the behaviour [70]. That is Level C evidence obtained with an antibody rather than a particle, and it has not been repeated with a multivalent carrier. Read with the calibrated-flow observation that surface-available HER2 did not correlate with the internalised fraction of a conjugate across eight lines [33], it argues against treating receptor abundance, surface-accessible receptor and internalised carrier as one graded quantity. Fixed-tissue measurement reports the first; the delivery question concerns the second and the third.

### 3.5. Size, Shape, Curvature and Membrane Wrapping

Receptor-mediated uptake requires the membrane to wrap the particle, consuming receptors from the surrounding membrane. Continuum analysis identified an optimal radius minimising wrapping time, with uptake becoming prohibitively slow for particles too small to provide sufficient adhesion energy or too large to recruit enough receptors [71]. Gold nanoparticles of 14, 50 and 74 nm gave uptake half-lives of 2.10, 1.90 and 2.24 h [72], and antibody-coated particles of 40–50 nm produced the greatest effect on receptor internalisation and signalling [73]. Shape modifies adhesion under flow, with nanorods showing higher specific and lower non-specific accumulation than spheres against vascular endothelium [74]; that work concerns endothelial targets under flow and suggests, but does not establish, an equivalent advantage against tumour-cell antigen.

These parameters interact with antigen density rather than acting independently of it. Comparing targeted and untargeted gold particles across sizes, an advantage for active targeting appeared only at 60 nm and was absent at 15, 30 and 100 nm (Level D) [75]. A geometry that maximises wrapping in a receptor-rich membrane may be a poor choice in a receptor-poor one, since the recruitable receptor pool is what limits wrapping.

### 3.6. Is There a Minimum HER2 Density for Particulate Targeting?

Among the studies identified, one addressed this question directly with receptor abundance as the independent variable (Level A). Using HER2-targeted liposomal doxorubicin across a graded panel of HER2 expression, nuclear doxorubicin delivery increased with receptor number and showed a threshold effect at approximately 2 × 10^5^ receptors per cell, which kinetic modelling attributed to multiple low-affinity liposome–receptor interactions; human cardiomyocytes express HER2 below this level, consistent with the absence of cardiomyocyte uptake [36].

That result is central and should be read carefully. It is a platform-specific, experimentally observed engagement threshold for one HER2-targeted liposomal system, not a general property of HER2 targeting, and it is the only measurement of its kind we identified. Placing its numerical value is less straightforward than it appears. Against the second calibrated determination, 2 × 10^5^ receptors per cell sits above T-47D (8203) and MDA-MB-453 (47,050) and below BT-474 (277,096) and AU-565 (456,584) [34], and appears to separate weakly from strongly expressing lines; against the first it does not, since there MDA-MB-453 was measured at 2.75 × 10^5^ and falls above the value while every HER2-positive line exceeded 10^6^ [33]. One cell line therefore lands on either side of it depending on which calibrated assay is consulted. Neither determination separates clinical categories, because no validated mapping exists between an IHC score and a receptor-per-cell value (Section 2.5), and whether tumours reported as HER2-low reach this level of surface-accessible receptor is not established.

Clinical experience with the same construct cautions against reading that value as a criterion for benefit. The liposome from which it was derived entered a randomised trial in patients classified as HER2-positive and was stopped early for futility (Section 6.1) [37,38]. The defensible inference is narrow: clinical HER2 positivity was not sufficient to guarantee benefit from that particular regimen. It does not demonstrate failure of active targeting, insufficient receptor abundance or a transport bottleneck; regimen composition, dose and exposure, carrier design, payload, the comparator arm, patient selection and unmeasured tumour biology are all compatible with the result, and no data discriminate among them.

The measurement carries several constraints. It describes one platform, with one ligand chemistry, one ligand density and one payload; it was made in vitro, with nuclear drug delivery rather than clinical efficacy as the readout, and it is expressed as receptor abundance rather than true surface density. Threshold position would in any case be expected to shift with ligand valency and per-bond affinity, with the steric environment of the polymer corona, with particle size, and with receptor mobility and clustering [40,46,47,51,75]. We identified no antigen-density cut-off applicable across particulate platforms. Engagement thresholds appear platform-specific, must be measured for the carrier in question, and cannot be inferred from clinical HER2 categories or used for patient selection. Figure 1 sets the categorical scale and the measured values side by side without aligning them.

### 3.7. How Carrier Properties Move the Apparent Density Requirement

If an engagement threshold is a property of a carrier–receptor pair rather than of HER2, the carrier variables that set it are worth stating together. The preceding subsections introduced them separately; their joint effect is that the receptor abundance a given design requires is not a fixed number but a quantity that moves with the design. The direction of each effect is reasonably well established. The magnitude, at low receptor abundance, generally is not, because almost none of these parameters have been varied against quantified receptor abundance.

Diameter acts at two stages at once. It governs extravasation and interstitial penetration, with 30 nm micelles penetrating a poorly permeable xenograft that 50–100 nm micelles entered only after vascular permeability was raised pharmacologically [77], and vascular normalisation improving delivery of 12 nm particles while hindering that of 125 nm particles in the same model [78]; it governs membrane wrapping and the number of ligands that can be displayed, with an optimal radius predicted by continuum analysis [71] and an active-targeting advantage observed at 60 nm but not at 15, 30 or 100 nm in a matched comparison (Level D) [75]. Geometry modifies adhesion under shear and the amount of protein adsorbed, with elongated particles showing higher specific and lower non-specific accumulation against vascular endothelium [74] and high-curvature features reducing corona thickness [79]. Surface charge acts mainly on non-specific interactions rather than on receptor engagement: gold particles of differing charge altered plasma membrane potential in malignant and non-malignant cells [80], and a graded charge series built on one micellar scaffold identified a slightly negative surface as optimal for tumour delivery [81]. All three parameters are therefore established as determinants of delivery and unestablished as determinants of the density requirement.

The ligand layer is where the density requirement is set most directly, and where nominal specification is least informative. Only a minority of conjugated ligands remain functionally accessible, a twentyfold increase in conjugated antibody yielding approximately a twofold increase in functional binding sites [56], so a carrier described by nominal ligand density is not characterised for this purpose. Tether length and brush architecture then determine whether the ligand present can reach the receptor at all: peptides tethered on a long spacer and buried within the liposomal poly(ethylene glycol) brush gave no uptake advantage over untargeted liposomes, whereas shortening both brush and tether so that the ligand projected beyond the brush raised uptake approximately ninefold in breast cancer cells and about a hundredfold in myeloma cells, uptake plateauing near 2% peptide density in both [53]. Attachment chemistry and orientation govern whether that accessibility survives contact with plasma, non-covalent pre-adsorption outperforming covalent coupling [82] and oriented coupling through a scaffold preserving a several-fold targeting advantage in undiluted plasma [83], while corona formation can abolish ligand function outright [84,85]. Ligand mobility acts in the same direction on fluid interfaces, where lateral rearrangement of bound components shifts the binding transition to lower receptor densities (Level E) [46,47].

What these parameters jointly determine is effective valency: the number of bonds a particle can actually form on contact, which is smaller than the ligand count and depends on receptor density, ligand accessibility, contact geometry and membrane deformability. Effective valency with per-bond affinity then sets avidity, and free-energy computation of antibody-bearing carriers predicted a surface coverage below which apparent affinity collapses, validated against endothelial ICAM-1 [50], while particles bearing a bimodal brush selected for receptor density through a steric barrier surmountable only by many simultaneous bonds [51]. Two consequences bear on how this literature should be read. An engagement threshold quoted for one carrier does not transfer to another, and the two parameters most often reported, nominal ligand density and hydrodynamic diameter, are among the least sufficient to specify one. Section 11 sets out the individual trade-offs, and the point here is that they are not independent: a change made to improve one stage of the cascade routinely moves the apparent density requirement at another.

One order-of-magnitude estimate is worth stating, because the two quantities it needs are now available and because it makes effective valency concrete. Combining the detected surface densities of Section 3.4 with the projected contact area of a sphere, a 100 nm carrier overlays of the order of one receptor on the highest-density line measured and roughly a fifth of that at 50 nm, while within a cluster of the reported radius and occupancy the local density is more than an order of magnitude above the cell mean [62]. The estimate is ours rather than the source’s, and three assumptions bound it: localisation microscopy reports detected molecules, so the densities are lower bounds; projected area understates the contact area once membrane wrapping begins; and the measurement samples the adherent membrane of cultured cells. The direction is nonetheless unambiguous, and it is not the one usually assumed. At the densities measured, multivalent attachment cannot be a property of the initial contact footprint alone; it requires receptors to be recruited into the contact zone during particle residence, which makes lateral mobility and pre-existing clustering (Section 3.4) and the wrapping mechanics of Section 3.5 rate-determining rather than incidental. Lower still, the same estimate places no receptor at all under much of the initial footprint, which is the physical content of the threshold behaviour reported for one liposomal system [36] and of the superselective prediction [40].

## 4. The Moving Target: Spatial and Temporal HER2 Heterogeneity

### 4.1. Intratumoural Heterogeneity

Mean expression is a poor descriptor of a surface a carrier samples locally. Spatial transcriptomic profiling of HER2-positive tumours found significant differences in ERBB2 expression between cancer-cell clusters within individual patients [76], and multiplexed imaging resolves tumour-cell phenotypic structure at single-cell resolution [86]. Quantified heterogeneity carries prognostic weight: among 251 HER2-positive patients, a biphasic distribution of HER2 gene copy number gave ten-year disease-free survival of 63% against 91% for monophasic tumours (*p* < 0.001) [87]. We identified no equivalent quantitative work specifically in HER2-low tumours.

For delivery, the distributional question is sharper than the prognostic one. Two tumours with identical mean HER2 signal behave differently if one presents a uniform field of moderately expressing cells and the other a receptor-rich minority in a receptor-poor background; in the second, a superselective carrier would bind the rich subpopulation preferentially, and that preferential binding is itself an obstacle to reaching the remainder (Section 8).

Three quantities are conflated when heterogeneity is discussed, and they are worth separating here as elsewhere. Histological heterogeneity is what pathology reports: variation in staining intensity or in the proportion of stained cells, ordinal and reader-dependent. Genomic heterogeneity is what single-cell in situ methods report; in 164 patients given neoadjuvant T-DM1 with pertuzumab, heterogeneity defined as ERBB2 amplification in more than 5% but fewer than 50% of tumour cells, or a HER2-negative area by fluorescence in situ hybridisation, was present in 16 of 157 evaluable cases and was accompanied by a pathological complete response rate of 0% against 55% in non-heterogeneous tumours [88]. Quantitative surface-accessible receptor heterogeneity, the distribution across cells of the receptor a carrier can engage, is what the delivery argument requires, and we identified no study reporting it in human tumours. The first two inform the third without substituting for it, since amplification heterogeneity need not be proportional to surface-protein heterogeneity and neither is measured on live membrane.

Where a below-threshold subpopulation exists, the consequences extend beyond the immediate distribution of delivered dose. Selective delivery to receptor-rich cells is selection pressure, and in situ single-cell analysis of HER2-positive breast cancer through neoadjuvant treatment found treatment-associated changes in the spatial distribution of cellular genetic diversity that correlated with poor outcome after adjuvant trastuzumab [89]. For a superselective carrier, the same logic applies more sharply, because the delivered-dose distribution approaches a step rather than a gradient, and payload properties then determine whether the spared fraction is reached at all; the quantitative evidence for neighbour-cell exposure remains Level C from conjugates (Section 9.6) [90,91,92]. This is a mechanistic expectation rather than an observation: no particulate system has been shown to select for a HER2-low subpopulation in vivo, and the experiment has not been reported.

### 4.2. Interlesional Discordance

Discordance between primary tumour and metastasis is substantial and bidirectional. Among 547 matched primary–relapse pairs, overall HER2 discordance was 38.0%, comprising 15% HER2-0 converting to HER2-low and 14% the reverse [13]; in 232 matched primary–metastasis pairs agreement was fair at best (κ = 0.33; 95% CI 0.21–0.44), with 44% of HER2-zero primaries scoring higher on biopsy and 22% of HER2-low primaries becoming IHC 0 [93]; a cohort of 3834 women reported 38.8% discordance in recurrent cases [14].

Whole-body imaging shows lesion-level variation prospectively and at whole-body scale, although it reports a composite rather than receptor abundance alone. In ZEPHIR, 93 of 265 lesions (35%) across 42 of 90 patients were HER2-negative on ^89^Zr-trastuzumab positron emission tomography (PET) despite HER2-positive histology [94,95], and quantitative analysis of 264 evaluable lesions found up to eightfold variation within a single patient and thirteenfold between patients [96]. These figures describe heterogeneity in tracer behaviour—target biology together with accessibility, delivery and retention—rather than a direct measure of receptor-expression heterogeneity (Section 10.2).

### 4.3. Temporal Evolution

HER2 expression is not fixed across a treatment course: in DAISY it decreased at resistance in 13 of 20 evaluable patients [42]. Part of the discordance in retrospective series is analytical rather than biological, since the same specimens disagree between readers [22] and assays [27]; we identified no study separating true conversion from measurement noise, so serial assessment must hold both explanations open.

### 4.4. Consequences for Particulate Delivery

Selecting patients for a receptor-directed carrier from an archival primary tumour is unreliable when roughly a third of paired samples disagree, and from a single contemporaneous biopsy when lesions within one patient differ up to eightfold by imaging. Where a carrier binds superselectively, spatial heterogeneity would additionally be converted into a near-binary distribution of delivered dose, making the fraction of cells above the engagement threshold, rather than mean expression, the descriptor that matters.

## 5. What Antibody–Drug Conjugates Teach Us, and What They Cannot

The clinical activity of T-DXd in HER2-low disease is why antigen density at the low end has become interesting, and the Level C comparison with ado-trastuzumab emtansine (T-DM1) is informative. It is also frequently over-extended.

### 5.1. What the ADC Comparison Establishes

T-DM1 carries a non-cleavable maleimidomethyl cyclohexane-1-carboxylate linker at a drug-to-antibody ratio of approximately 3–3.6, with its preclinical activity confined to HER2-overexpressing cells [97]. T-DXd carries a cathepsin-cleavable tetrapeptide linker at approximately 7.7, with plasma payload release of only 1.2–3.9% at day 21; it was active in patient-derived xenografts scored IHC 1+ without amplification in which T-DM1 was not, and a ratio of 8 was active in low-expression models where 3.4 was not [98].

Payload membrane permeability is likely to be a major contributor to this difference, although the two conjugates also differ in linker chemistry, drug-to-antibody ratio, payload potency, intracellular processing and pharmacokinetics, and the cited work does not partition the effect between them. The exatecan derivative has a log D of 2.3 against 0.2 for the lysine-linked maytansinoid catabolite, an effective permeability of 12.2–13 × 10^−6^ cm/s against less than 0.1 × 10^−6^ cm/s, and a half-maximal inhibitory concentration against HER2-negative cells of 0.5 nM against 40.5 nM [90]. In the experimental systems studied, cytotoxicity tracked released payload more closely than target expression or conjugation ratio, and membrane-permeable payloads produced bystander killing where non-permeable ones did not [91].

Bystander killing is local and diffusion-limited: in a separated-inoculum design only the co-inoculated antigen-negative tumour responded [90], and spatiotemporal separation of antibody and payload showed it only partially breaches the binding-site barrier [99]. Higher conjugation ratios are not free either; increased loading accelerates clearance roughly threefold and lowers the maximum tolerated dose, so equal antibody doses of differently loaded conjugates were comparably effective [100].

Clinically, the predictive value of measured HER2 within the low range remains weak. In DAISY, confirmed objective response was 29.7% (95% CI 15.9–47) in tumours scored IHC 0, with responses in 5 of 14 patients below and 3 of 10 above median ERBB2 expression [42], and extended DESTINY-Breast04 follow-up reported efficacy consistent across IHC status [3]. Outside the HER2-low range, EMILIA showed similarly limited discrimination by tumour HER2 messenger RNA in a HER2-positive population, progression-free survival hazard ratios above and below the median being essentially identical (0.65 versus 0.64) [101]. Within currently available clinical datasets, HER2 abundance in the low range has shown limited predictive discrimination for ADC benefit.

### 5.2. Why This Does Not Validate Particulate Targeting

An immunoglobulin G molecule has a hydrodynamic radius of 5.29 nm [102]; a therapeutic liposome or polymeric nanoparticle is typically 50–200 nm. Tumour vascular permeability falls steeply with size: interstitial penetration at 30 min fell from more than 35 µm for 3.3–10 kDa tracers to approximately 5 µm for a 2 MDa tracer, maximal accumulation and maximal penetration occurring at different sizes [103], and active targeting of gold particles conferred an accumulation advantage at one diameter only [75].

Mechanistic modelling incorporating antigen concentration predicted that, for liposome-sized objects, antigen targeting will not substantially increase tumour uptake [104]. The most direct test remains the immunoliposome work: targeted and untargeted long-circulating liposomes reached comparable tumour concentrations of approximately 7–8% of the injected dose per gram, while targeting raised intracellular uptake in cancer cells by up to a factor of six, and in non-HER2-overexpressing MCF-7 xenografts the targeted liposomes accumulated in stroma without cancer-cell internalisation [105]. Ligand decoration altered cellular disposition after arrival rather than increasing total accumulation.

Delivery is heterogeneous for antibodies themselves, which bounds how much of the ADC experience can be borrowed. At the clinical dose, about 10% of tumour cells were reached by T-DM1 in a gastric xenograft, and co-administering unconjugated trastuzumab at 3:1 to 8:1 doubled the survival gain by improving distribution [106]. Larger multivalent carriers should not be assumed to distribute more homogeneously.

The comparison is informative on payload chemistry and on the limits of bystander killing. It is not evidence that a HER2-targeted particulate carrier will function in HER2-low disease, because the two agent classes differ in the steps that antigen density constrains.

### 5.3. Receptor-Density Requirements of Conjugates and Particulate Carriers Compared

The two agent classes are commonly discussed as though they differed mainly in size. They differ in how each stage of the delivery cascade scales with antigen density, and setting the comparison out explicitly determines how much of the ADC experience can be carried across.

Transport scales against the particle. An immunoglobulin G molecule has a hydrodynamic radius of 5.29 nm and is bivalent [102]; a therapeutic liposome or polymeric nanoparticle is 50–200 nm and carries tens to hundreds of ligands. Effective diffusivity falls with molecular radius [107], and vascular permeability falls steeply with size over the same range [103], so antibody-format agents move through tumour interstitium more readily than particles one to two orders of magnitude larger. Particles are additionally cleared by the mononuclear phagocyte system rather than salvaged by the neonatal Fc receptor, and are sequestered by tumour-associated macrophages once in tissue [108,109]. The consequence is that a particle is commonly transport-limited before it can be binding-limited, whereas an antibody at conventional dose is characteristically binding-limited near the vessel, which is what the binding-site barrier describes [110,111].

Binding scales the other way. An antibody’s retention is governed by monovalent or bivalent affinity and is approximately proportional to receptor availability over the relevant range; a particle’s retention is governed by the collective off-rate of many weak contacts and can depend on receptor density in a strongly non-linear manner (Section 3.1) [40]. A conjugate should therefore continue to bind and deliver as antigen falls, inefficiently but continuously, while a multivalent carrier operating in a superselective regime may lose engagement over a comparatively narrow density interval. That contrast is a prediction from Level E physics applied to one Level A observation in a single liposomal system [36,40], not a demonstrated property of clinical carriers.

Payload stoichiometry differs by orders of magnitude, and in the opposite direction to valency. A conjugate carries approximately 3–8 payload molecules per antibody [97,98,100], so cytotoxicity depends on repeated binding, internalisation and intracellular accumulation; measured with three orthogonal assays in high- and low-HER2 lines, intracellular exposure to unconjugated payload, total drug and total antibody was substantially higher in the high-HER2 line, and model-based sensitivity analysis identified internalisation rate, degradation rate, HER2 expression and efflux as the determinants [112]. A drug-loaded liposome carries orders of magnitude more payload per carrier, so a single productive internalisation event delivers a much larger intracellular bolus and fewer successful engagements per cell are needed. Bystander killing is a property of the released payload rather than of the carrier [90,91] and is in principle available to both classes, but it has been quantified only for conjugates, and the local, diffusion-limited character demonstrated there bounds what either class can expect [99].

Set out this way, the ADC result in HER2-low disease constrains the particulate question in one direction only. It establishes that a receptor present at levels the diagnostic assay was not built to quantify can support therapeutically useful delivery by a small bivalent agent carrying a membrane-permeable payload. It does not establish that the same receptor abundance supports multivalent capture of an object one to two orders of magnitude larger that must first extravasate, cross interstitium and escape phagocyte sequestration, nor that the density dependence of the two processes has the same shape. The one direct comparison available, in which targeted and untargeted liposomes reached comparable tumour concentrations while targeting altered only cellular disposition [105], indicates that the particulate case turns on a different rate-limiting step.

## 6. HER2-Directed Particulate Platforms: What the Evidence Supports

### 6.1. The One Platform Tested Clinically

MM-302, a PEGylated liposomal doxorubicin bearing an anti-HER2 single-chain variable fragment, is the most clinically advanced HER2-targeted particulate platform we identified. In a phase 1 study of 69 patients with HER2-positive disease, the maximum tolerated dose was not reached; among patients treated at 30 mg/m^2^ or above, objective response was 13% and median progression-free survival was 7.4 months, with a 28% response rate in the 25 anthracycline-naive patients [113]. The randomised phase 2 HERMIONE trial tested that regimen against physician’s choice chemotherapy [37] and was terminated after 113 patients; the registry entry citing a data monitoring committee view that benefit over control was not shown, confirmed by futility analysis [38]. No results are posted, and no peer-reviewed efficacy publication exists, so the trial supports only the inference set out in Section 3.6: HER2 positivity did not guarantee benefit from this regimen, and it cannot identify which of the regimen-, carrier-, payload-, transport- or selection-level explanations applies.

The companion imaging substudy is the most informative human dataset we identified. Using ^64^Cu-labelled MM-302 in 19 patients, all classified as HER2-positive by conventional criteria, lesion deposition at 24–48 h varied approximately 35-fold (0.52–18.5%ID/kg of lesion, a unit not interchangeable with the %ID/g used for preclinical biodistribution), was independent of plasma exposure, and was associated with more favourable outcomes where higher [39]. A comparable qualitative pattern appeared with an untargeted agent, ferumoxytol magnetic resonance imaging in mixed advanced solid tumours, finding exposure differing 8.3-fold across all imaged lesions and 1.03- to 4.22-fold within individual patients [114]. Both are cited throughout as independent illustrations that lesion-level transport can vary by an order of magnitude in patients, including in tumours uniformly classified as HER2-positive. They are not a head-to-head comparison: agents, carrier chemistries, imaging modalities, denominators, tumour populations and study designs all differ, and the values are not commensurable.

### 6.2. Preclinical Platforms Across Material Classes

The preclinical literature spans liposomes, polymeric particles, lipid nanoparticles, protein cages, dendrimers, inorganic and hybrid systems, and engineered extracellular vesicles. Its aggregate message is more uniform than its material diversity suggests. Table 2 sets out the studies central to the mechanistic argument; three patterns stand out.

Ligand format has been examined more carefully than receptor abundance. Holding the particle constant and comparing trastuzumab (150 kDa), a designed ankyrin repeat protein (14 kDa) and an affibody (8 kDa), the small scaffolds allowed 10- to 40-fold more ligands per particle, yet selectivity for HER2-positive over HER2-negative cells stayed between approximately 2.3- and 2.8-fold for all three [115]—higher valency raising binding without proportionally raising discrimination, as expected when receptor rather than ligand availability is limiting.

Control design is inconsistent. The stronger studies use a matched untargeted particle and, better, an isotype-matched conjugate: multivalent trastuzumab half-chains on iron oxide compared against both bare and IgG-conjugated particles [116]; affibody-decorated mesoporous particles compared with an identical untargeted particle, with blockade-confirmed receptor-mediated uptake [117]; and one dendrimer study using an isogenic pair differed only in HER2 transfection, the cleanest design for isolating receptor abundance [118]. Where an untargeted comparator is absent, an efficacy result cannot be attributed to the ligand [119]. Extracellular-vesicle platforms enter on the same terms, with an oligoclonal nanobody-decorated exosome having been reported to bind selectively and deliver doxorubicin in HER2-positive models [120].

Receptor abundance is rarely quantified in absolute terms. Across this literature, HER2 was reported as percentage of positive cells, mean fluorescence intensity or immunoblot signal, and conversion to receptors per cell was performed in only one study identified [36]. Most work therefore sits at Level B, so claims of the form “effective in a HER2-positive model” are not comparable across studies and cannot be positioned relative to clinical HER2-low disease.

**Table 2:** Selected HER2-targeted particulate drug-delivery systems central to the mechanistic argument of this review. The studies were chosen for the step of the delivery cascade each informs, and the table is not an inventory of the field. The relevance column gives the evidence level defined in Section 3 with a one-line statement of what each study can and cannot support. Only one of the directly relevant studies identified in the literature reviewed here met the Level A definition, which illustrates the limited direct evidence base. Evidence levels describe the type of source, not study quality, certainty of evidence, risk of bias or clinical efficacy. The table has been expanded to state, for every entry, the targeting architecture and the design parameter it varies, the method by which HER2 was characterised, whether receptor abundance was actually quantified, and whether density dependence was tested. Where a study did not quantify receptor abundance, this is stated as such, and no receptor-per-cell value has been inferred from a categorical HER2 label or from an immunohistochemical score. The additional entries characterise carrier-design variables—ligand format and valency, functional ligand density, steric accessibility, particle size, corona state, triggered release and dual targeting—and the receptor-level consequences of multivalent engagement.

Platform and Targeting Architecture	Model or Population	HER2 Quantification Method	Receptor Abundance reported; Density Dependence Tested	Principal Finding (Delivery or Biological Endpoint)	Evidence Class, Relevance and Principal Limitation	Ref.
PEGylated liposome (MM-302)	Phase 1, 69 patients with HER2-positive metastatic breast cancer (efficacy figures from the ≥30 mg/m^2^ subset)	Clinical IHC/ISH; not quantified	Not quantified. Density dependence not tested	MTD not reached; scFv terminal half-life 63.3 h; at ≥30 mg/m^2^, ORR 13% and median PFS 7.4 months; ORR 28% in the 25 anthracycline-naive patients	Level B; clinical, HER2-positive only; receptor abundance not quantified	[113]
PEGylated liposome (MM-302), randomised	Randomised phase 2 versus physician’s choice chemotherapy, HER2-positive disease; 250 planned	Clinical IHC/ISH	Not quantified. Density dependence not tested	Design and endpoints per protocol [37]; terminated after 113 enrolled, registry citing no demonstrated benefit confirmed by futility analysis [38]	Level B; clinical, HER2-positive only; receptor abundance not quantified	[37,38]
^64^Cu-labelled liposome (MM-302)	19 patients with HER2-positive metastatic breast cancer	Clinical IHC/ISH; deposition quantified by PET	Not quantified; carrier deposition quantified instead. Density dependence not tested	Lesion deposition varied ~35-fold (0.52–18.5%ID/kg), independent of plasma exposure; higher deposition associated with better outcome	Level B; the only HER2-targeted carrier imaged in patients. Deposition quantified, receptor abundance not	[39]
Long-circulating immunoliposome	BT-474 (HER2-overexpressing) and MCF-7 (non-overexpressing) xenografts	Comparative, not calibrated	Not quantified. Density dependence tested categorically only, as overexpressing versus non-overexpressing	Targeted and untargeted liposomes reached comparable tumour levels (~7–8%ID/g); targeting increased intracellular uptake up to sixfold; in the non-overexpressing model the targeted liposomes accumulated in stroma without cancer-cell internalisation	Level B; targeting benefit shown to be expression-dependent in vivo, but the comparator is an uncalibrated non-overexpressing line, not a HER2-low tumour	[105]
HER2-targeted liposomal doxorubicin	Graded panel of HER2 expression, cell-level delivery	HER2 quantified as receptors per cell	Quantified as receptors per cell across a graded panel. Density dependence tested; threshold at approximately 2 × 10^5^ receptors per cell	Nuclear doxorubicin delivery showed a threshold at ~2 × 10^5^ HER2 receptors per cell, attributed to multiple low-affinity interactions	Level A; the only antigen-density titration for a HER2-targeted particle identified here, and the source of the platform-specific threshold discussed in Section 3.6	[36]
Gold nanoparticle with defined ligand number	BT-474, MCF-7 (moderate HER2), MDA-MB-468; in vivo MCF-7	Comparative across three expression levels	Not quantified in molecules per cell; three expression levels compared. Density dependence tested categorically	Two antibodies were superior in vitro; one antibody gave approximately twofold greater tumour accumulation in vivo	Level B; in vivo work in a moderate-HER2 model with ligand number controlled exactly; expression not calibrated	[58]
Liposome, ligand-density series	Spheroids	Ligand density calibrated; receptor density not	Not quantified; single expression level. Density dependence not tested	Targeted formulations outperformed plain liposomes, but differences across the twofold ligand-density range (330 to 690 antibodies per particle) were not statistically significant	Level B; quantifies functional ligand density, single expression level, in vitro	[55]
Superparamagnetic iron oxide	Cells of differing receptor density	Receptor density varied experimentally	Not quantified in molecules per cell; receptor density varied experimentally. Density dependence tested, uncalibrated	Intermediate ligand density outperformed both higher and lower densities; optimum conserved across particle size and lower-receptor-density cells	Level B; varies ligand and receptor density, but expression not calibrated to molecules per cell	[52]
Lipid nanoparticle (untargeted)	HER2-low patient-derived xenograft; HER2-negative cell-derived xenograft	Model selected as HER2-low	Not quantified; model selected as HER2-low. Density dependence not tested	Restoring HER2 and p53 expression sensitised previously unresponsive tumours to trastuzumab	Supporting HER2-low model evidence; outside the A–E targeting hierarchy, because the carrier itself is untargeted. Premised on such tumours not being addressable by conventional HER2-targeting	[41]
Magnetic nanocarrier, three ligand formats	HER2-positive and HER2-negative cell lines in vitro	Comparative cell-line classification; not calibrated	Not quantified. Density dependence not tested; ligand format and valency varied instead	Small scaffolds permitted 10- to 40-fold more ligands per particle, yet selectivity for HER2-positive over HER2-negative cells remained approximately 2.3- to 2.8-fold for all three formats	Level B; isolates ligand format at constant particle, indicating that higher valency raises binding without proportionally raising discrimination. Limitation: two categorical expression levels, in vitro only	[115]
Iron oxide with multivalent trastuzumab half-chains	HER2-positive breast cancer cells and xenografts	Comparative; not calibrated	Not quantified. Density dependence not tested	Multivalent display improved antitumour potential and reduced resistance relative to both bare and IgG-conjugated particles	Level B; one of few studies including an isotype-matched comparator, which separates multivalent display from HER2 engagement. Limitation: HER2-positive models only	[116]
Mesoporous nanoparticle with dimeric affibody	HER2-positive tumour models	Comparative; not calibrated	Not quantified. Density dependence not tested	Affibody decoration increased uptake and chemo-radiotherapeutic effect over an identical untargeted particle, with blockade confirming receptor-mediated uptake	Level B; blockade control establishes receptor mediation. Limitation: single, high expression level	[117]
Poly(amidoamine) dendrimer, VHH-redirected	Isogenic cell pair differing only in HER2 transfection	Isogenic comparison; not calibrated	Not quantified. Density dependence tested only as presence versus absence of receptor	Delivery and cytotoxicity were HER2-dependent in cells differing only in HER2 expression	Level B; the cleanest design identified here for isolating receptor dependence from other cell differences. Limitation: binary rather than graded expression	[118]
Nanobody-decorated exosome	HER2-positive breast cancer models in vitro and in vivo	Comparative; not calibrated	Not quantified. Density dependence not tested	Oligoclonal nanobody decoration gave selective binding and doxorubicin delivery in HER2-positive models	Level B; extends the platform range to engineered extracellular vesicles on the same terms as synthetic carriers. Limitation: HER2-positive models only, no graded expression series	[120]
Nanoparticle with single-molecule counting of functional ligand	Cell lines spanning a range of receptor density, in vitro	Single-molecule quantification of ligand; receptor density compared across lines	Not quantified in molecules per cell. Ligand functionality correlated with cell receptor density	Only a minority of conjugated fragments remained functionally accessible, a twentyfold increase in conjugated antibody producing approximately a twofold increase in functional binding sites	Level B; the basis for distinguishing functional from nominal ligand density throughout this review. Limitation: binding rather than payload-effect endpoint, in vitro	[56]
Lipid nanoparticle with quantified functional ligand, anti-CD8	T cells in vivo; target receptor is CD8, not a tumour antigen	Not applicable to HER2	Not quantified in molecules per cell. Ligand density varied; performance peaked at low functional ligand density	In vivo performance peaked at low ligand density, and higher densities degraded the target receptor	Level D; shows that excess ligand carries a cost beyond waste, including degradation of the target. Limitation: non-HER2 receptor on a non-tumour target cell	[57]
Peptide-targeted liposome, PEG and linker series	HER2-overexpressing breast cancer cells and VLA-4-expressing myeloma cells in vitro	Comparative; not calibrated	Not quantified. Density dependence not tested; ligand steric accessibility varied instead	Ligand buried within the PEG brush gave no advantage over untargeted liposomes, whereas shortening both brush and linker so that the ligand projected beyond the brush raised uptake approximately ninefold and about a hundredfold in the two models; uptake plateaued near 2% peptide density	Level B and D; the clearest demonstration identified here that steric accessibility rather than ligand number can determine whether targeting works at all. Limitation: in vitro, single expression level per model	[53,54]
Gold nanoparticle size series, targeted versus untargeted	Tumour xenografts in vivo, four diameters	Comparative; not calibrated	Not quantified. Density dependence not tested; particle diameter varied instead	An advantage for active targeting appeared at 60 nm only and was absent at 15, 30 and 100 nm	Level D; shows that the benefit of targeting is conditional on particle size. Limitation: non-HER2 receptor, accumulation rather than efficacy endpoint	[75]
Enzyme-activated HER2-targeted liposome	Ovarian xenograft in vivo	Comparative; not calibrated	Not quantified. Density dependence not tested	Combined HER2 targeting with a matrix-metalloproteinase-cleavable stealth polymer and outperformed both non-targeted and non-cleavable controls	Level B; reduces reliance on receptor-mediated internalisation by de-shielding on site. Limitation: single high expression level, ovarian rather than breast model	[121]
Ultrasound-triggered trastuzumab immunoliposome	HER2-positive and HER2-negative cells in vitro	Comparative; not calibrated	Not quantified. Density dependence tested categorically, HER2-positive versus HER2-negative	Triggered release raised uptake several-fold in HER2-positive but not in HER2-negative cells	Level B; an informative negative, since a trigger acts on carriers already present and cannot substitute for the binding step. Limitation: binary expression comparison, in vitro	[122]
Dual-ligand liposome and dual-targeted lipid nanoparticle	Breast cancer cell lines in vitro	Comparative; not calibrated	Not quantified. Density dependence not tested, although uptake tracked HER2 expression in one of the two studies	A trastuzumab/iRGD liposome improved half-maximal inhibitory concentration by roughly 13–15% over the better single-ligand formulation, without in vivo confirmation and with greater haemolysis; a HER2/folate lipid nanoparticle exceeded single-targeted formulations across three lines while uptake still tracked HER2 expression	Level B; where matched single-ligand arms are included the gains are modest. Limitation: in vitro, no graded receptor series, no matched-arm in vivo comparison	[123,124]
Transferrin-functionalised nanoparticle, corona study	Cell lines in vitro, in serum-containing medium	Not applicable to HER2	Not applicable. Density dependence not tested; corona state varied instead	Targeting capability was lost once a biomolecule corona formed, while non-specific cellular entry continued	Level D; establishes that ligand function on arrival rather than ligand presence at formulation is what matters. Limitation: non-HER2 receptor, in vitro	[84]
Antibody-engineered magnetofluorescent nanocrystal	HER2-positive tumour-bearing mice	Comparative; not calibrated	Not quantified. Density dependence not tested	Internalisation proceeded through complex formation with HER2 and produced substantial downregulation of surface HER2 protein	Level B; shows that multivalent engagement modifies the target itself, a self-limiting behaviour of particular concern where receptor is scarce. Limitation: HER2-positive model, downregulation reported qualitatively	[125]
Untargeted iron oxide agent (ferumoxytol), magnetic resonance imaging	Patients with mixed advanced solid tumours	Not assessed	Not applicable; no receptor targeting. Density dependence not tested	Lesion exposure differed 8.3-fold across all imaged lesions and 1.03- to 4.22-fold within individual patients	Outside the A–E targeting hierarchy because the carrier is untargeted; cited as an independent human illustration that lesion-level transport varies by an order of magnitude. Limitation: not commensurable with the targeted-carrier imaging dataset	[114]

Abbreviations: CD8, cluster of differentiation 8; HER2, human epidermal growth factor receptor 2; %ID/g, percentage of injected dose per gram; %ID/kg, percentage of injected dose per kilogram; IgG, immunoglobulin G; IHC, immunohistochemistry; iRGD, internalising RGD peptide; ISH, in situ hybridisation; MTD, maximum tolerated dose; ORR, objective response rate; PEG, poly(ethylene glycol); PET, positron emission tomography; PFS, progression-free survival; scFv, single-chain variable fragment; VHH, single-domain antibody variable region; VLA-4, very late antigen-4. Evidence levels A–E are defined in Section 3.

### 6.3. What Is Missing

We identified no study comparing a HER2-targeted particle against its matched untargeted counterpart across a graded panel of quantified HER2 expression in vivo with survival endpoints. In its absence, the antigen-density dependence of particulate delivery rests on three indirect sources: cell-level titration [36]; binding physics, one study varying receptor density on a synthetic interface and the other ligand coverage on the carrier [50,51]; and the one in vivo comparison in which a targeting advantage present in a HER2-overexpressing model was absent in a non-overexpressing one [105].

## 7. The Preclinical Model Problem

Most HER2-targeted particulate systems are evaluated in BT-474, SK-BR-3, SK-OV-3 or NCI-N87 and their xenografts; the lines bead-calibrated flow cytometry places in the 10^6^ range (Section 2.5) [33]. What survives both determinations is the spread: the standard models sit one to two orders of magnitude above the weakly expressing lines, and above the platform-specific engagement threshold reported for one HER2-targeted liposome [36].

Demonstrating that a HER2-decorated particle binds HER2, and that binding improves uptake in a cell expressing 10^6^ receptors, answers a different question from the one posed by HER2-low disease. Two claims are frequently conflated: that a platform can engage HER2, and that it remains effective as HER2 abundance falls. Only the second is translationally relevant, and it is rarely tested.

Model limitations extend beyond receptor number. Patient-derived xenografts acquire mouse-specific copy-number evolution across passages [126], and subcutaneous xenografts differ from human tumours in vascular architecture, stroma and immune context. Most consequentially, the enhanced permeability and retention phenomenon on which passive accumulation depends is more pronounced and more uniform in rodent models than in patients, as the human imaging discussed in Section 6.1 indicates [39,114]; clinical transport heterogeneity has therefore been proposed as a major limitation of nanomedicine translation [127]. A model reproducing neither the receptor abundance nor the transport heterogeneity of the disease cannot adjudicate between target and carrier limitation.

The assumptions that license extrapolation are worth naming, because they are what a reader is asked to grant. Extrapolating from a HER2-overexpressing line to HER2-low disease assumes that the mechanism observed is continuous in receptor abundance and does not change regime, an assumption the superselectivity prediction specifically contradicts (Section 3.1) [40]. Extrapolating from a weakly expressing line used as a HER2-low surrogate assumes that its receptor abundance corresponds to that of the clinical category, which cannot be verified because no validated mapping between IHC score and receptor number exists (Section 2.5) and because the same line differs by close to sixfold between calibrated determinations [33,34]. Extrapolating from a non-HER2 receptor assumes shared trafficking and spatial organisation, which for HER2 is specifically uncertain given its atypical endocytic handling (Section 3.4) [65,68,69]. Extrapolating from a synthetic interface assumes that a rigid, chemically defined surface bearing a known receptor density is a fair proxy for a deformable membrane with clustered, mobile receptors and a glycocalyx. Extrapolating from a rodent xenograft assumes transport behaviour that human carrier imaging does not support [39,114,127]. And extrapolating from human imaging assumes that tracer behaviour reports receptor availability, when it reports a composite (Section 10.2). None of these assumptions is unreasonable; all of them are assumptions, and the evidence-level labels used throughout mark which one is being made at each point.

Receptor abundance should be reported as a calibrated quantity with the assay named, and a platform proposed for HER2-low disease should be tested in at least one model in the relevant abundance range, patient-derived where available [41].

## 8. When Targeting Fails: Locating the Bottleneck

Failure of a receptor-directed particulate system has more than one cause, and which one applies determines the response: patient and lesion selection, a change in ligand architecture, reformulation, or a change in payload.

### 8.1. Steps at Which Delivery Can Fail

Figure 2 maps the cascade, marking where antigen density is directly limiting, where transport, clearance and carrier surface state predominate, and where successful binding itself becomes an obstacle.

Arrival. Quantitative surveys place median tumour delivery at 0.7–0.76% of the injected dose [128,129], a contested metric [130]; delivery is dose-dependent, and saturating hepatic macrophage uptake raised it to as much as 12%, reaching 93% of tumour cells [131].

Entry. Entry appears dominated by active transendothelial transport rather than passive inter-endothelial gaps, with direct counting finding 26 gaps across 313 vessels and modelling attributing as much as 97% of 50 nm gold particle accumulation to the transcellular route [132], with macropinocytosis proposed as the mechanism [133]. That capacity is unevenly distributed: 83.7% of vessels contain no particle clusters [134], and much of what accumulates later exits through lymphatics [135].

Reaching a tumour cell. Accumulation in tissue is not delivery to tumour cells: quantitative accounting at 24 h placed an upper bound of 0.0014% of the injected dose on the fraction reaching cancer cells, no more than about two of every hundred being contacted [136]. Tumour-associated macrophages held roughly 30% of intratumoural nanoparticles, their depletion lowering drug concentration by about a quarter [108], and hepatic clearance accounts for much of the remainder [109].

Surface competence on arrival. Serum protein adsorption can abolish ligand function: transferrin-functionalised particles lost targeting once a biomolecule corona formed while still entering cells non-specifically [84], and targeting significant in healthy plasma was abolished by tumour-bearing plasma in one model [85]. Conjugation chemistry and ligand orientation modulate the effect (Section 9.8) [82,83].

Binding without penetration. The binding-site barrier was described analytically for antibodies, binding retarding percolation into tissue while high affinity worsens distribution rather than improving it [110], and shown experimentally in a guinea-pig carcinoma where penetration was severely hindered at conventional doses and only partly overcome by a 33-fold dose increase [111]. Transient competitive inhibition improved trastuzumab distribution from 26.5% to 43.3% of tumour area, but total uptake fell by more than 15% once inhibitor persistence exceeded a threshold [137,138]. All of this is Level C work on antibodies and conjugates; whether an equivalent barrier operates for particles, transport-limited well before they are binding-limited, is untested.

Interstitial transport. Size governs how far a particle moves once extravasated: in a poorly permeable pancreatic xenograft only 30 nm micelles penetrated effectively, and 50–100 nm micelles did so only when vascular permeability was raised pharmacologically by transforming growth factor-β inhibition [77]. Such constraints lie largely upstream of tumour-cell HER2 engagement and are not determined by receptor density alone, although what is retained and taken up on arrival is not similarly independent of it.

**Figure 2:**
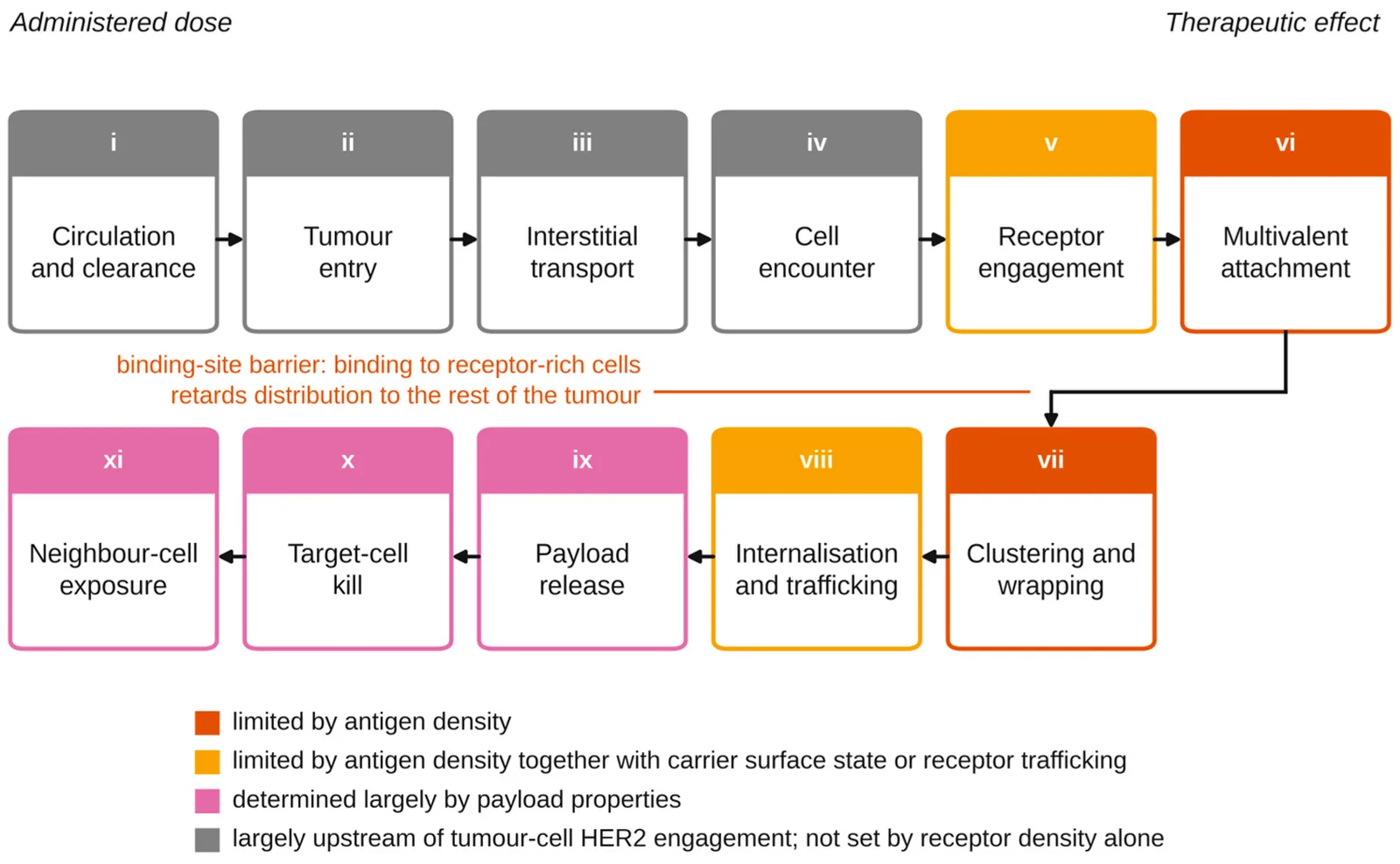
Where antigen density acts in the particulate delivery cascade, and where other constraints predominate. Each stage of the cascade is coloured by the factor expected to limit it. Vermilion: limited by antigen density: multivalent attachment, which in superselective architectures may show threshold-like loss of engagement below a given density [36,40,51], and receptor clustering with membrane wrapping, which consumes receptors from the surrounding membrane [71]. Amber: limited by antigen density together with carrier surface state or receptor trafficking: ligand–receptor engagement, at which corona formation may already have abolished ligand function [84,85], and internalisation with trafficking, governed by recycling and efflux as well as receptor number [65,67]. Pink: determined largely by payload properties: release, target-cell killing and neighbour-cell exposure, the last demonstrated for conjugates rather than particulate carriers [90,91,99]. Grey: lying largely upstream of tumour-cell HER2 engagement and not determined by receptor density alone: circulation and phagocyte clearance [109,131], tumour entry [132,133,134], interstitial transport [77,103] and cell encounter [136]. These map onto the operational classes of Section 8.2, the grey and amber stages corresponding to the two carrier classes and the pink stages to payload limitation. The annotation marks the binding-site barrier, the origin of distribution limitation, characterised for antibodies and conjugates only [110,111,137]. The figure is conceptual, carries no quantitative axes and assigns no relative weights.

Perfusion, matrix and pressure. Upstream of every step above lies whether a given tumour region is perfused at all, and at what pressure. Elevated interstitial fluid pressure flattens the transcapillary pressure gradient on which convective transport depends, leaving transport into tumour interstitium diffusion-dominated [107,139]. The interstitial matrix then sets the diffusion resistance: across four tumour types, resistance to macromolecular transport tracked total collagen content and tissue stiffness rather than glycosaminoglycan content, and collagenase treatment increased immunoglobulin diffusion [140]. The two barriers respond differently to intervention, and favour opposite designs. Vascular normalisation lowered interstitial fluid pressure and improved delivery of 12 nm particles while impairing delivery of 125 nm particles in the same model, because the reduction in pore size that lowers pressure also raises steric resistance [78]; modelling of combined vascular normalisation and solid-stress alleviation places the optimum for intratumoural distribution at agents of 10 nm or less with low binding affinity [141]. Both findings point away from the size range of most HER2-targeted particulate platforms.

Penetration distance is the quantity these barriers act on. Doxorubicin concentration measured against CD31-stained vasculature fell approximately exponentially with distance from vessels, halving at roughly 40–50 µm, while mean distance from blood vessels in the same tumours was 90–140 µm, so many viable tumour cells received no detectable drug after a single injection [142]. That measurement concerns a small molecule, which diffuses far more readily than a particle; it is small-molecule and general particulate transport evidence outside the A–E targeting hierarchy, cited as an upper bound on what a larger carrier could achieve rather than as particulate evidence; reviews of nanomedicine transport reach the same conclusion from the vascular and interstitial abnormalities directly [143]. The implication for the present argument is asymmetric and worth stating plainly. Adequate surface-accessible HER2 cannot rescue a carrier that does not reach the cells expressing it, whereas a carrier that does reach them may still fail for want of receptor. The two constraints are not interchangeable, and a platform has to satisfy both.

### 8.2. Target Limitation Versus Carrier Limitation

These failure modes include two broad upstream categories, target limitation and carrier limitation. Target limitation means the accessible receptor is insufficient, or too heterogeneously distributed, to sustain multivalent engagement for a given carrier; its signatures are loss of the targeted-versus-untargeted difference in cellular uptake at preserved tissue accumulation [105], threshold behaviour on titration against quantified receptor number [36], and preferential binding to receptor-rich subpopulations. Carrier limitation means the carrier does not arrive, does not retain functional ligand, or cannot move through tissue, and separates operationally into a transport-and-clearance class, signalled by low or heterogeneous tissue accumulation despite a shared clinical HER2-positive classification [39] and by macrophage-dominated distribution [108] or size-dependent penetration failure [77], and a surface-state class, signalled by loss of ligand function in plasma [84,85]. Two further classes act downstream of arrival and binding. Distribution limitation means binding succeeds but is confined to receptor-rich or perivascular cells, characterised for antibodies and conjugates [110,111,137]. Payload limitation means enough carrier arrives and binds, but the payload cannot kill the cells reached or their antigen-poor neighbours [90,91,92]. Figure 3 therefore distinguishes five operational limitation classes, and sets out how to tell them apart.

Two published observations warn against assuming target limitation by default. Surface-available HER2 measured by calibrated flow cytometry across eight lines did not correlate with the proportion of conjugate internalised, and lines at both ends of the range showed cytotoxicity [33]. Separately, the one HER2-targeted nanoparticle taken into a randomised trial failed to show benefit in a population uniformly classified as HER2-positive [37,38], which excludes categorical receptor status as a sufficient explanation of success without identifying the limiting step.

The distinction is practical, because each class implicates a different design variable. Target limitation is addressed by patient and lesion selection, by ligand architecture, or by abandoning receptor-mediated internalisation. The two carrier classes are addressed by formulation and by conjugation chemistry respectively, and no amount of receptor abundance will rescue either. Distribution limitation is addressed by per-ligand affinity, valency and dosing schedule, and payload limitation by potency, membrane permeability and release site. A study reporting only tumour-level accumulation cannot separate them, which is why cellular-resolution readouts and matched untargeted comparators are the minimum needed to interpret a negative result.

### 8.3. From Receptor Binding to Biological Response

Receptor abundance, receptor occupancy, internalised carrier number, intracellular payload amount and pharmacodynamic effect are five distinct quantities. The argument of this review concerns the first; the clinical endpoint concerns the last. The links between them are neither fixed nor, for particulate carriers, quantified, and conflating them is how a plausible mechanistic chain acquires unwarranted precision.

Occupancy is not a stable readout even for antibodies. Its measured value depends on assay design, with free-receptor versus bound-receptor formats and baseline-normalised versus timepoint-normalised calculations diverging substantially when the internalisation rate of bound receptor exceeds the degradation rate of free receptor [144], which is the regime an internalising targeted receptor occupies. No universal receptor-occupancy requirement for therapeutic effect has been established for HER2, and we identified none proposed for a particulate carrier. That analysis is Level E by the scheme of Section 3, which is a quantitative-systems-pharmacology treatment of a different receptor, and it is cited for the measurement problem it identifies rather than for any HER2-specific value.

Nor is greater occupancy monotonically better at tissue level. Saturating the receptors encountered first is what produces the binding-site barrier, and in a HER2 xenograft co-administering unconjugated trastuzumab at 3:1 to 8:1 with a fixed T-DM1 dose—deliberately lowering occupancy per cell in order to distribute the conjugate across more cells—approximately doubled the improvement in median survival, with payload delivered per cell in vivo already exceeding the amount required for efficacy [106]. Where that holds, the limiting quantity is the number of cells reached rather than the amount delivered to each, and maximising binding at the receptor-rich fraction is counterproductive.

What determines killing, in the systems where it has been measured, is the intracellular amount of released payload rather than target expression or conjugation ratio. Conjugates directed at different receptors and carrying identical payload showed cytotoxicity tracking released intracellular payload concentration independently of receptor level or drug-to-antibody ratio [91], and cell-level pharmacokinetic measurement in high- and low-HER2 lines identified internalisation, degradation, expression and efflux as the determinants of intracellular exposure [112]. We identified no study establishing the number of internalised particles, or the intracellular payload concentration, required for cell kill by a HER2-targeted particulate carrier, and no such threshold should be assumed from conjugate data, since payload per carrier, release kinetics and intracellular routing all differ. The chain from binding to response is therefore intact as a qualitative account and open at every quantitative link. All of the quantitative evidence in this subsection is Level C, obtained with conjugates rather than particulate carriers, which is why this review states a threshold only where one was measured, and states it as a property of the platform on which it was measured.

## 9. Engineering Around Low HER2 Density

Every strategy below trades one limitation for another, and the test that matters is whether it preserves delivery as receptor abundance falls, a comparison rarely reported.

### 9.1. Optimising Rather than Maximising Ligand Density

Section 3.3 found intermediate optima, a large gap between nominal and functional ligand number and, in two systems, a penalty for excess [52,55,56,57,58]. Whether the optimum moves when receptor becomes scarce is unsettled: superselectivity would predict an upward shift, while the penetration and clearance penalties of high ligand density are unaffected by receptor abundance. We identified no study titrating ligand density against a graded receptor series in vivo, so the recommendation stays procedural—titrate against the intended receptor range and report functional ligands per particle.

### 9.2. Affinity Engineering

Section 3.2 sets out why lowering per-ligand affinity improves distribution for antibody-format agents. Modelling of high-avidity, low-affinity carriers placed the optimum dissociation constant in the low nanomolar range while noting that the predicted advantage diminishes at 10^4^–10^5^ receptors per cell [145]—the range spanned by the weakly expressing lines used as HER2-low surrogates, so the advantage may be smallest where most wanted. Such antibodies have since been tested with HER2 conjugates in vivo, improving penetration in high-expression tumours with limited loss of uptake in low-expression ones (Level C) [146]. Among the studies identified, none showed the same for a nanoparticle; the strategy is mechanistically motivated rather than validated.

### 9.3. Multivalent Architecture and Ligand Geometry

Section 3.4 sets out the Level E evidence that nanoscale ligand spacing can matter as much as ligand number [59,60,61]. Geometric control would in principle raise engagement efficiency without raising ligand number, attractive where receptor is scarce, but precise geometry is hard to impose on soft, polydisperse carriers and the scaffolds permitting it carry a pharmacokinetic penalty: a pH-gated ligand-display construct clearing rapidly through the kidney without selective tumour accumulation when given intravenously [147].

### 9.4. Dual-Targeted and Complementary-Target Designs

Adding a second ligand distributes the binding requirement across two receptor populations, but where matched single-ligand arms are included, the gains are modest: a dual trastuzumab/iRGD liposome improved half-maximal inhibitory concentration by roughly 13–15% over the better single-ligand formulation, without in vivo confirmation and with greater haemolysis [123], and a dual HER2/folate lipid nanoparticle exceeded single-targeted formulations across three cell lines yet uptake still tracked HER2 expression [124].

Three qualifications bound what this strategy can be expected to achieve where HER2 is scarce. Distributing the binding requirement across two receptor populations lowers the density of each that is needed only if both ligands can engage simultaneously from one particle footprint, which depends on the spatial coincidence of the two receptors and not merely on their co-expression; none of the studies identified measured that. A heterobivalent design also divides the available surface between two ligands at fixed total ligand density, so the gain is not free, and where matched single-ligand arms were included, the measured gains were modest [123,124]. And a ligand directed at a microenvironmental rather than a tumour-cell target, such as matrix, vasculature or infiltrating myeloid cells, changes where a carrier is retained rather than which cells internalise it, and therefore addresses transport limitation rather than target limitation (Section 8.2). The alternative to distributing the requirement is to remove it by installing an artificial receptor [148] or restoring expression with a nucleic-acid payload [41], each of which relocates the selectivity problem upstream rather than solving it (Section 9.5 and Section 9.7). We identified no study comparing a dual-targeted carrier with its matched single-targeted counterpart across a graded, quantified receptor series, which is the experiment the claim requires.

### 9.5. Reducing Dependence on Receptor-Mediated Internalisation

If internalisation is the step that fails at low receptor density, extracellular release is a rational alternative. Liposomes combining HER2 targeting with a matrix-metalloproteinase-cleavable stealth polymer outperformed both non-targeted and non-cleavable controls in ovarian xenografts [121], while ultrasound-triggered release from trastuzumab immunoliposomes raised uptake several-fold in HER2-positive but not HER2-negative cells [122]—an informative negative, since a trigger acts on carriers already present and cannot substitute for the binding step. A more radical version installs an artificial receptor by metabolic glycoengineering [148], relocating the selectivity problem to precursor delivery rather than removing it.

### 9.6. Payload Properties and Neighbour-Cell Exposure

Where a fraction of tumour cells lies below the engagement threshold, payload properties determine whether those cells are reached. Membrane-permeable payloads produce neighbour-cell killing and non-permeable ones do not [90,91], and linker–payload engineering nearly suppressed the antigen-negative population in a heterogeneous xenograft [92]. The mechanism is local, cannot substitute for distribution [99], and raises exposure of normal neighbours; all of it is Level C evidence from conjugates, with no quantitative demonstration for a particulate carrier identified.

### 9.7. Creating or Modulating the Target

Expression can be modified rather than targeted as found: lipid nanoparticle-delivered messenger RNA restoring HER2 and p53 sensitised HER2-low and HER2-negative xenografts to trastuzumab [41]. Pharmacological upregulation is less straightforward than assumed, since in gastric models class I histone deacetylase inhibition downregulated HER2 irrespective of baseline expression while HER2 kinase inhibition upregulated it in low-expressing cells [149]. Any priming strategy therefore needs direct measurement of surface receptor, not transcript.

### 9.8. Preserving Ligand Function in Biological Fluid

Corona formation can abolish ligand function outright [84], and the effect depends on plasma composition [85]. Mitigations with primary support include non-covalent pre-adsorption of antibody, which outperformed covalent coupling [82]; oriented coupling through a scaffold, preserving a several-fold targeting advantage in undiluted plasma [83]; and high-curvature geometry, which reduced corona thickness [79]. Cell-membrane camouflage deserves scrutiny, since a quantitative assay found up to 90% of biomimetic particles only partially coated [150,151]. None of these was tested at low receptor density in the studies identified.

Bypassing the binding-site barrier is a related problem. Transient competitive inhibition improves distribution at a measurable cost in total uptake [137,138], and size reduction improves penetration in poorly permeable tumours [77]; tumour-penetrating peptide co-administration is widely cited but did not survive an independent replication [152]. All the bypass strategies identified were validated in high-expression models, where the barrier is strongest, and whether they help at low antigen density is untested.

### 9.9. Receptor Crosslinking, Signalling and Effects Beyond Payload Delivery

A multivalent carrier does something to the receptor as well as to the cell. Engaging many receptors simultaneously is, physically, crosslinking, and crosslinking a receptor tyrosine kinase is not a neutral act. This matters in two directions: it may be exploitable, and it confounds the interpretation of a targeted-versus-untargeted comparison.

For HER2 the evidence is suggestive and incomplete. Efficient cross-linking relocated ErbB2 out of membrane protrusions into bulk membrane, coated pits and endosomes, which, in that account, is the event that renders an internalisation-resistant receptor internalisation-competent [68]. Antibody-coated gold particles of 40–50 nm produced the greatest effect on receptor internalisation and downstream signalling, the effect depending on particle size rather than on the antibody alone [73]. Antibody-engineered magnetofluorescent nanocrystals delivered to HER2-positive tumours in mice were internalised through complex formation with HER2 and produced substantial downregulation of surface HER2 protein [125], and multivalent display of trastuzumab half-chains on iron oxide particles improved antitumour effect relative to both bare and isotype-conjugated particles [116]. Level D and E work in other receptor systems show the principle more cleanly: tight nanoscale clustering of Fcγ receptors on nucleic-acid scaffolds promoting phagocytosis [59] and hexagonal Fas-ligand arrays raising signalling efficiency by two orders of magnitude over soluble ligand [60].

Three consequences follow. A carrier may exert an antiproliferative or pro-apoptotic effect not attributable to its payload, which is why an untargeted particle carrying the same payload is a necessary but not a sufficient control. An isotype-conjugated particle holds multivalent protein display constant and isolates what HER2 engagement adds (Section 6.2) [116]; separating the biology of crosslinking from the action of the payload requires the further comparison of a ligand-bearing, payload-free particle, which is reported less often than it should be. Induced downregulation of the target reduces the receptor available to subsequent doses, a self-limiting behaviour already observed for excess ligand density in a non-HER2 lipid nanoparticle system [57] and of particular concern where baseline receptor is scarce. And because crosslinking-dependent internalisation requires a minimum number of simultaneous bonds, these consequences would themselves be expected to be antigen-density-dependent, and therefore least available in the setting where they would be most useful. None of the three has been tested across a graded, quantified HER2 series.

## 10. From HER2 Classification to Quantitative Targetability

### 10.1. What Tissue-Based Quantification Now Provides

Calibrated tissue assays exist and disagree with categorical scoring in informative ways. Quantitative immunofluorescence traceable to mass spectrometry resolves HER2 in the unamplified range and places most unamplified tumours above its limit of quantification [30,31]; targeted mass spectrometry quantifies HER2 in fixed tissue [153] and separates IHC 2+/ISH-negative from 2+/ISH-positive tumours with an area under the curve of 0.908 [154]; and calibrated quantitative immunohistochemistry reports receptor number per cell over a range that does not reach the ultralow region (Section 2.5) [34]. All report fixed-tissue abundance. Reproducibility can be improved without changing that: digital image analysis raised agreement against expert consensus (weighted κ 0.84 versus 0.76) and separated ultralow from null tumours with high specificity in a single-centre series [155]; artificial-intelligence assistance raised agreement between two pathologists reading 209 slides from κ 0.610 to κ 0.713 in HER2-non-amplified cases [24]; and continuous optical-density scoring stratified T-DXd benefit better than categorical scoring in an exploratory trial analysis [156].

The strongest evidence identified here that continuous measurement may provide discrimination beyond categorical scoring comes from a multi-platform analysis of a T-DXd-treated cohort in which quantitative immunofluorescence, evaluable in 51 patients, was continuously associated with time to next treatment (HR 0.77 per 5-unit increment; 95% CI 0.68–0.86) while IHC categories performed sub-optimally [157]. That analysis was retrospective, the quantitative subset small and uncorrected for multiple comparisons, so superiority over categorical scoring is not established prospectively and replication is required.

### 10.2. What Imaging Provides and What It Does Not

Whole-body HER2 imaging addresses lesion-level and temporal sampling in a way biopsy cannot: affibody PET separated HER2-positive from HER2-negative lesions without overlap [158], ^89^Zr-trastuzumab PET has resolved lesion-level discordance with histology [94,95], and quantitative analysis has put inter- and intra-patient heterogeneity in tracer behaviour at up to thirteen- and eightfold respectively [96].

Performance within the low-expression range is another matter. In HER2-low patients, tracer uptake did not reach a significant correlation with IHC score (r = 0.41; *p* = 0.24), an underpowered negative rather than a demonstration of no relationship [159]. In paired imaging–biopsy analysis, maximum standardised uptake value was higher in HER2-low than in HER2-positive lesions and did not reliably separate the two [160]; quantitative ^89^Zr-trastuzumab PET/magnetic resonance failed as a response predictor (area under the curve 0.47) [161]; and earlier work in HER2-negative primaries found false-positive lesions with higher uptake than true positives [162]. HER2 PET uptake reflects a composite of receptor abundance, tracer delivery, accessibility and retention. That composite is precisely what makes it informative about whole-body target and delivery heterogeneity, and precisely what limits its use as a direct surrogate for receptor abundance.

For particulate delivery, the more pertinent imaging is of the carrier itself. Radiolabelled liposome PET and ferumoxytol magnetic resonance imaging both quantified order-of-magnitude inter-lesional variation in patients (Section 6.1) [39,114], the latter also predicting colocalisation of a therapeutic nanoparticle in mice [163]. We identified no prospective trial selecting patients for nanoparticle therapy on such a measurement.

### 10.3. Analytical Performance and Comparability of HER2 Quantification Methods

Section 2.4 described the reproducibility of categorical scoring and Section 10.1 the calibrated assays that improve on it. What has not been set out together is what each method measures, how it performs analytically, and whether it reports the quantity this review argues is operative. Table 3 does so, and three distinctions drawn there are worth stating in the text.

The first is between an ordinal and a continuous measurand. Chromogenic immunohistochemistry returns one of four categories from a visual read, and its behaviour at the 0 versus 1+ boundary—26% reader concordance in one study, 68.9% inter-laboratory concordance overall falling to 57.6% for slides scored IHC 1+—is a property of that measurand rather than of careless reading [22,24]. Digital image analysis and artificial-intelligence assistance improve agreement without changing what is measured [24,155], and continuous optical-density scoring converts the same stain into a graded variable [156]. Calibrated quantitative immunofluorescence, calibrated quantitative immunohistochemistry and targeted mass spectrometry return continuous values in defined units, and are the only tissue methods identified here that do [30,31,34,153,154].

The second is between analytical and clinical performance, which are established separately and neither of which implies the other. Limit of detection, limit of quantification, dynamic range, linearity and reproducibility are analytical characteristics of an assay; whether a value above or below a given point identifies patients who benefit is a clinical question answered only by prospective testing against an outcome. The strongest available signal that continuous measurement carries information beyond the category—a hazard ratio of 0.77 per 5-unit increment of quantitative immunofluorescence for time to next treatment, in 51 evaluable patients, retrospective and uncorrected for multiple comparisons [157]—is an analytically continuous measurement whose clinical validation is still outstanding. The distinction matters here because a platform-specific engagement threshold, if one were adopted for patient selection, would require both.

The third, and for this review the decisive one, is between tissue abundance and surface accessibility. Of the methods in Table 3, only bead-calibrated flow cytometry on unpermeabilised cells and single-molecule localisation microscopy with an extracellular-domain probe report receptor availability to an extracellular binder, and both require live or freshly prepared cells rather than an archival block [33,62]. Every method applicable to a diagnostic specimen reports fixed-tissue abundance or signal. A quantitative assay is therefore not, by virtue of being quantitative, a measurement of the pool a carrier engages, and describing one as such would overstate what it delivers.

### 10.4. Single-Cell and Spatial Technologies: What They Can and Cannot Measure

Methods that resolve heterogeneity at cellular scale are the obvious candidates for characterising the distribution this review argues is decisive. What they contribute depends entirely on which molecule they detect, and the distinction is routinely blurred.

Spatial transcriptomics and single-cell RNA sequencing measure ERBB2 transcript: not HER2 protein, and not protein at the plasma membrane. Spatial profiling of HER2-positive tumours found significant differences in ERBB2 expression between cancer-cell clusters within individual patients [76], which is informative about the architecture of expression. It is not a measurement of surface-accessible receptor, and the step from transcript to protein is not reliable enough to be treated as one. Transcript abundance is an incomplete predictor of protein level across systems [164], and for HER2 specifically, an independent comparison of reverse-transcription PCR-based measurement against protein and gene-amplification assays found a high false-negative rate, with only a minority of locally HER2-positive cases being called positive by transcript [165]. That comparison used a bulk commercial transcript assay rather than a single-cell or spatially resolved method, so it bounds the transcript-to-protein step in general rather than for these platforms specifically. Transcriptomic measurements are valuable here for what they are, and should be described as such.

Spatially resolved protein methods come closer to the quantity of interest and still fall short of it. Imaging mass cytometry images tens of proteins at subcellular resolution on tissue sections [166], and multiplexed tissue imaging has been qualified for breast cancer and applied at scale, with cyclic immunofluorescence across 567 HER2-positive samples from 189 patients segmenting 201,601 single cells and identifying tumour-cell clusters of heterogeneous HER2 expression co-varying with other receptors [167]. Multiplexed imaging also resolves the phenotypic structure of the microenvironment, including the myeloid populations that sequester particulate carriers [86,108]. These methods deliver the cell-to-cell distribution of HER2 protein in spatial context, which is what a delivery argument needs—measured, however, on fixed tissue and without discriminating surface from intracellular receptor.

The gap that remains is therefore narrow and specific. Single-molecule localisation microscopy with an extracellular-domain probe does report surface receptor per unit membrane area together with its clustering, and has been applied to freshly excised patient tissue by touch preparation [62], but it has not been developed into a validated tissue assay, reported in the HER2-low range, or related to any delivery endpoint. What a density-guided strategy would require is a method combining that surface restriction with the spatial resolution and tissue compatibility of the multiplexed platforms. We identified none in clinical use, and note that the gap is technological rather than conceptual.

### 10.5. What a Density-Guided Strategy Would Require of a Companion Diagnostic

If targetability is platform-specific and has to be measured, a carrier developed on that premise implies a companion measurement, and the requirements are more demanding than those met by any current HER2 assay.

The regulatory category itself is established. The United States Food and Drug Administration defines an in vitro companion diagnostic device as one providing information essential for the safe and effective use of a corresponding therapeutic product, and its final guidance states that the diagnostic and the therapeutic should generally be approved or cleared contemporaneously [168]; draft guidance sets out principles for co-development of the two and, as issued, carries non-binding recommendations [169]. HER2 supplies the precedent: approval of trastuzumab deruxtecan for HER2-low and HER2-ultralow hormone receptor-positive disease was accompanied by approval of a single immunohistochemical assay, tied to one antibody clone, as a companion diagnostic [6]. That precedent is instructive in what it required—one assay, one clone, one scoring convention, anchored to the trial population—and in what it did not: no calibration to receptor abundance and no measurement of surface accessibility.

Four requirements would distinguish a density-guided particulate companion diagnostic from that precedent. Analytical validation would have to be established for a continuous measurand across the low range rather than for a categorical call at a boundary, with stated limits of detection and quantification, dynamic range, precision and inter-laboratory reproducibility (Table 3). Clinical validation would have to establish a cut-off, and by the argument of Section 3.6 that cut-off would be platform-specific, so assay and carrier would need to be co-developed rather than the assay adapted afterwards; a cut-off validated for one carrier would not transfer to another, which is a development constraint as much as a scientific one. Sampling would have to address inter-lesional discordance, since a single biopsy is a poor estimator where 38% of matched primary–relapse pairs are discordant [13] and lesion-level tracer behaviour varies up to eightfold within one patient [96]—an argument for whole-body assessment, tempered by the fact that the imaging signal is a composite rather than a receptor measurement (Section 10.2). And the assay would have to report the surface-accessible pool, which no clinically validated method currently does (Section 2.5 and Section 10.3).

Four consequences follow for present practice, and they are worth separating because they are often collapsed into one. For threshold interpretation, a value measured on one quantity cannot be applied to another: the engagement threshold discussed in Section 3.6 is expressed as receptor abundance and was measured in vitro, and no clinical specimen records that quantity, so the number cannot be read against a pathology report. For patient selection, it follows that no currently available assay identifies the patients whose tumours a receptor-directed carrier would productively engage, and that categorical HER2 status is not a substitute for one (Section 2.5 and Section 3.6). For retrospective biomarker analysis, an archived block does not record the live surface pool, so an absent association between HER2 category and carrier benefit in such a dataset would not discriminate between insufficient accessible receptor and insufficient delivery. And for prospective trial design, a density-guided trial cannot yet be designed as such; what can be designed is one that measures carrier deposition as an endpoint rather than as a selection biomarker, which is already feasible in patients [39,114] and would establish whether such a measurement predicts anything before it is used to select anyone (Section 12).

We make no prediction about which regulatory pathway such a device would follow, since that depends on the claim made and on jurisdiction, and the available guidance addresses co-development in general rather than receptor-density-guided nanomedicine in particular. The point is narrower: the measurement problem described in Section 2.5 is not solved by adopting a quantitative assay, and any carrier whose development assumes a selection biomarker should state which quantity that biomarker reports.

### 10.6. A Conceptual and Operational Framework

We identified no single number predicting whether a given carrier will work in a given tumour, nor a validated model of this kind in the literature reviewed here. What the evidence supports is a decomposition into separately measurable terms: effective particulate targetability as the joint contribution of surface-accessible antigen density, spatial accessibility, binding competence, internalisation competence and payload effectiveness. This is a conceptual framework, not a mathematical model, and no coefficient or functional form is implied. Its usefulness is that each term corresponds to an experiment: antigen abundance to calibrated tissue quantification, which needs live-cell or surface-restricted methods for the accessible fraction [30,33,34]; spatial accessibility to imaging and spatial profiling [39,76,86]; binding competence to functional ligand quantification [56] and avidity measurement at defined receptor or ligand density [50,51]; internalisation competence to receptor-blocked uptake and trafficking kinetics [65,67]; and payload effectiveness to potency, membrane permeability and neighbour-cell exposure [90,91]. A platform that fails should be diagnosable to a term, which current reporting practice does not allow (Figure 3).

Existing mechanistic models are consistent with this decomposition and incorporate antigen concentration explicitly [104]. Their most relevant prediction, that for liposome-sized objects antigen targeting does not substantially increase tumour uptake, is supported by the immunoliposome biodistribution data in which targeted and untargeted carriers reached comparable tumour levels [105].

Stated as an operational sequence, the decomposition runs as follows, and every arrow marks a place where a measurement is possible and a coefficient is not available. Fixed-tissue HER2 abundance, which is what a diagnostic reports, bounds but does not determine the surface-accessible receptor density a carrier meets, and the ratio between them is unmeasured in human tumours (Section 2.5). Surface-accessible density, with receptor clustering and lateral mobility, determines the local density within a carrier footprint, which is what multivalent binding responds to rather than the cell mean (Section 3.4). Local density, functional ligand density, ligand accessibility and contact geometry determine effective valency; effective valency with per-bond affinity determines avidity and therefore retention, which in a superselective regime is a steeply non-linear rather than proportional function of density (Section 3.1 and Section 3.7). Retention permits internalisation, which is separately gated by receptor trafficking and is not proportional to binding [33,65,70]. Internalisation determines intracellular payload amount, which, with payload potency, release site, and membrane permeability, determines the effect on the cell engaged and on its neighbours (Section 8.3). At tissue level, the whole chain is weighted by the fraction of cells a carrier reaches at all, which transport determines independently (Section 8.1).

Each link in that sequence is supported by evidence; none has a validated coefficient or functional form in the HER2-low setting, and the chain has not been traversed end to end in any single system. Presenting it as an equation would therefore misrepresent the evidence, and we do not do so. What the sequence provides is an operational instrument: for a platform that underperforms, it specifies which link to interrogate and with what measurement (Figure 3), and it makes explicit that improving an upstream term cannot compensate for a broken downstream one. Section 10.7 sets out how far published quantitative models cover the same chain.

### 10.7. Quantitative and Computational Models: What They Specify and What They Omit

Published models address parts of that chain, and the useful question is which variables each contains, since a model that omits a variable cannot inform a design decision about it. Four families are relevant, and they do not overlap as much as their common subject suggests.

Multivalent binding theory is the most quantitatively developed segment. The superselectivity treatment computes bound fraction as a function of receptor surface coverage, ligand valency and per-bond free energy, and predicts the sharply non-linear dependence discussed in Section 3.1 [40]; it has been extended to probe design and to fluid interfaces with receptor clustering [44,46,47]. Free-energy computation of antibody-bearing nanocarriers incorporates ligand surface coverage, receptor density, per-bond affinity and the resistance of the endothelial glycocalyx; predicts the coverage below which apparent affinity collapses; and was validated against endothelial ICAM-1 in vitro, in vivo and by atomic force microscopy [50]. These models are quantitative, parameterised and, within their domain, tested. None was parameterised for HER2 on a tumour-cell membrane.

Membrane-wrapping models address internalisation. Continuum analysis returns an optimal particle radius from the balance of adhesion energy against membrane bending, uptake becoming prohibitively slow outside that range [71], and a related analytical treatment adds a non-specific interaction term, concluding that it contributes as strongly as specific binding and bond elasticity and returning threshold and optimal radii in closed form [170]. The broader theoretical and simulation literature treats wrapping as adhesion against bending rigidity and interaction range, including cooperative wrapping of several particles [171]. Their common limitation for the present purpose is that adhesion is represented as a smeared interfacial energy rather than as a countable set of receptor–ligand bonds, so receptor density enters implicitly if at all, and the receptor-depletion effect that matters at low abundance is not resolved.

Transport models address arrival and distribution, and are the family that carries antigen concentration most explicitly. The antibody penetration framework reduces biodistribution to diffusion opposed by systemic and antigen-mediated clearance, represents antigen concentration as antigen molecules per cell multiplied by cell density, and yields closed-form saturation timescales and the moving front that underlies the binding-site barrier [107,110]. The size- and affinity-dependent analysis introduced in Section 10.6 belongs to the same family and incorporates molecular size, binding affinity and antigen concentration [104,105]. Tumour-scale models of interstitial pressure and solid stress add the perfusion and matrix terms of Section 8.1 and locate the optimum for intratumoural distribution at agents of 10 nm or less with low binding affinity [141]. Physiologically based pharmacokinetic and multiscale frameworks extend this to whole-body disposition and to the size, shape, charge and corona parameters of Section 3.7, and reviews of the field describe agent-based and hybrid approaches alongside continuum ones [129,172,173].

Cell-level kinetic models close the chain at the other end. The threshold reported for one HER2-targeted liposome was itself the output of a kinetic model attributing the observed behaviour to multiple low-affinity liposome–receptor interactions [36]; internalisation rates have been modelled for HER2 across morphologically distinct cell states [66]; cellular processing rates for a trastuzumab–maytansinoid conjugate were determined and used to identify the design-sensitive parameters [67]; cell-level pharmacokinetic models fit intracellular antibody, total drug and released payload simultaneously and identify internalisation, degradation, expression and efflux as determinants [112]; and transport analysis of released payload has been used to predict the distance over which neighbour-cell killing operates [174].

Two observations follow. Coverage of the chain is uneven: binding and transport are well modelled, receptor trafficking and intracellular payload disposition less so, and no published model we identified spans surface-accessible HER2 abundance, ligand density, avidity, internalisation and biological response within one parameterised framework. And the assumptions do not hold uniformly across the range of interest, models being typically parameterised at receptor abundances of 10^5^–10^6^ molecules per cell, on cell lines, in rodent tumours whose transport properties are more favourable and more uniform than human ones [127,143], and with ligand density specified nominally rather than functionally [56]. A model built and validated under those conditions is a hypothesis-generating instrument in HER2-low disease rather than a predictive one, and none of those cited here has been prospectively validated against a clinical delivery endpoint; by the scheme of Section 3 this subsection is Level E throughout, with the transport and cell-level families additionally informed by Level C antibody and conjugate data. Their present value is to indicate which parameter a measurement should resolve, which is also the claim made for the framework in Section 10.6.

## 11. A Translational Design Framework

Targetability is a property of a combination, not of a label, and the three groups of variables that determine it are not independent.

Tumour variables comprise surface-accessible antigen density, its distribution within the lesion, the fraction of cells above the platform’s engagement threshold, inter-lesional variation, temporal stability under treatment, and microenvironmental transport, the last of which can be decisive (Section 6.1) [39,114]. Carrier variables—diameter, geometry, functional ligand density, ligand format and per-bond affinity, effective valency, corona behaviour and circulation kinetics—are expected to set the position of any engagement threshold, and thus which tumours fall above it [36,40,51]. Payload variables—potency, dependence on intracellular delivery, membrane permeability, release mechanism and neighbour-cell activity—may determine whether a tumour with a large below-threshold fraction is treatable at all, on Level C evidence from conjugates rather than particulate carriers [90,92].

A potent, membrane-permeable payload released extracellularly would be expected to reduce dependence on receptor-mediated internalisation and to broaden activity across heterogeneous expression, while one requiring lysosomal processing would be expected to do the opposite; the supporting evidence is Level C and drawn from conjugates rather than particulate carriers. Reducing particle size can improve interstitial penetration but may constrain ligand and payload capacity, and higher ligand density raises avidity, may reduce penetration, and can accelerate clearance or degrade the target receptor [57]. No configuration is optimal in general; the useful design question is which combination is viable within a specified antigen-density range.

Table 4 sets out these trade-offs parameter by parameter with the supporting evidence class, and Figure 3 addresses which variable is worth changing in an underperforming platform and what measurement would show it.

## 12. Research Priorities

The following are stated as measurements rather than aspirations.

1. Report calibrated receptor abundance, and move toward surface-accessible receptor. Studies should give molecules per cell with the assay named, in place of “HER2-positive”; the field also needs an assay reporting the surface-available fraction in tissue, which fixed-tissue methods do not provide [30,33,34].

2. Generate platform-specific antigen-density–response curves. Titrate delivery and efficacy against quantified receptor abundance for each carrier [36], reporting any threshold as a property of that carrier rather than of HER2.

3. Test in models spanning the clinically relevant low range. At least one model whose measured receptor abundance approaches that of weakly expressing tumours, patient-derived where possible [41].

4. Use heterogeneous-expression models, defined mixtures of receptor-rich and receptor-poor cells, with a cell-resolved readout of which population received payload.

5. Include matched untargeted, isotype and receptor-blocking controls, without which an efficacy result cannot be attributed to the ligand [105,116,117].

6. Quantify functional, not nominal, ligand density per particle, since the two differ by a large factor [55,56].

7. Report tissue-level and cell-level delivery separately, with the macrophage-associated fraction; the compartments dissociate [105,108,136].

8. Measure lesion-level deposition as an endpoint, not only as a selection biomarker. Carrier-deposition imaging is feasible in patients [39,114], but we did not find it reported alongside efficacy in the preclinical studies reviewed.

9. Quantify payload transfer to antigen-poor neighbouring cells for particulate carriers, as has been done for conjugates [92,99].

10. Titrate carrier design variables against a graded receptor series. Functional ligand density, effective valency, particle diameter, geometry and corona state should be varied against quantified receptor abundance within one system, so that any movement of an engagement threshold is measured rather than inferred [36,51,56].

11. Test density- or deposition-guided selection prospectively. No trial has selected patients for a particulate therapy on a quantitative receptor or carrier-deposition measurement; a feasibility study pairing such a measurement with a delivery endpoint would test the premise before an efficacy trial is made to depend on it [39,157].

## 13. Limitations of This Review

Several limitations bear on how the arguments above should be read; most describe the knowledge gap this review exposes rather than shortcomings of the synthesis, and they mark where inference ends. The literature selection was narrative and purposive, so statements of the form “we identified no study” describe this body of literature and are not proof of absence, and retrieval across three databases restricted to English will have excluded work indexed elsewhere. The evidence base is predominantly preclinical, and direct evidence in HER2-low or HER2-ultralow disease is close to absent: two human datasets report carrier deposition, only one with a HER2-targeted carrier [39,114]; one randomised trial of a HER2-targeted particle was conducted and terminated early [37,38]; and we identified no clinical experience of particulate HER2 targeting in HER2-low disease. Much of the argument therefore extrapolates across systems, signposted by evidence level throughout.

Several principles central to the argument—superselectivity, ligand spacing, the binding-site barrier—rest on Level D or E sources used as physical analogues rather than HER2-specific evidence, and the quantitative core rests in one case on a single in vitro titration [36], treated throughout as platform-specific rather than general or clinical. Reported receptor numbers are assay-dependent, obtained on cell lines, and differ by close to sixfold between determinations of the same line, so absolute values cannot be pooled onto a common scale. No validated conversion exists between an IHC category and a receptor-per-cell value, and we identified no clinically validated assay selectively quantifying surface-accessible HER2 in tumour tissue, so the quantity the argument turns on cannot currently be measured in patients. Nor did we identify an in vivo study comparing a HER2-targeted carrier with its matched untargeted counterpart across graded, quantified expression using clinically meaningful endpoints, or any prospective clinical validation of the framework proposed here. We also identified no published quantitative model spanning surface-accessible receptor abundance, ligand density, avidity, internalisation and biological response for a particulate carrier, so the framework proposed in Section 10.6 is an operational decomposition rather than a predictive model and is offered as such (Section 10.7).

## 14. Conclusions

HER2-low and HER2-ultralow are clinically meaningful categories that have changed treatment for a large population of patients. They are not measurements of target abundance, were not constructed to be, and the assay defining them is least reproducible precisely at the boundary that separates them.

For receptor-directed particulate delivery, what determines success is not whether HER2 is expressed but whether enough surface-accessible HER2 is present, in a spatial distribution compatible with carrier transport, for a specific carrier and payload to achieve productive delivery. That quantity is not yet routinely measurable in patients, because currently applicable calibrated tissue assays report fixed-tissue abundance rather than selectively quantifying the surface-accessible receptor pool. Antigen density is a genuine and frequently underappreciated constraint acting at several stages of the cascade, and it is not the sole one; whether it is dominant has not been determined, and the evidence assembled here does not permit that comparison. What the human imaging shows is narrower and still important: transport can remain a major bottleneck even where categorical clinical HER2 classification is held constant.

No antigen-density threshold generalisable across particulate platforms was identified in the literature reviewed here. The single measured value belongs to one liposomal system in vitro, cannot be mapped onto the clinical HER2-low category, and whether such tumours reach that level of surface-accessible receptor is not established. Successful particulate therapy depends jointly on target availability and its spatial distribution, carrier transport and surface functionality, intratumoural distribution, internalisation and trafficking, and payload properties. These determinants map onto the five operational limitation classes defined in Section 8.2, and failure at one level cannot necessarily be compensated for at another. Development should therefore move from categorical HER2 classification toward quantitative, platform-specific targetability, measured in experimentally realistic heterogeneous models and ultimately in patients rather than inferred from an immunohistochemical score.

Six questions determine whether the proposition advanced here can be converted into practice, and none of them is currently answered. They are stated as questions because the corresponding measurements, which are what would answer them, are listed in Section 12.

1. How should surface-accessible HER2 be quantified in patients? No clinically validated method reports the live, extracellularly available pool, and its relationship to fixed-tissue abundance is unmeasured in human tumours.

2. Are platform-specific engagement thresholds reproducible quantities? One has been measured, in vitro, for one liposomal system; whether such thresholds are stable properties of a carrier, and how they should be determined, is untested.

3. How far do carrier design variables move a threshold? Functional ligand density, effective valency, particle size, geometry and corona state are each expected to shift it, and none has been titrated against a graded, quantified receptor series.

4. Where does receptor availability stop being the limiting term? Transport, clearance and interstitial penetration constrain delivery independently of receptor abundance, and no measurement currently apportions a failure between them in patients.

5. How should a heterogeneous lesion be described for this purpose? The mechanism implies that the fraction of cells above a platform’s engagement threshold matters more than mean expression, and that fraction has never been measured.

6. Does density-guided selection change anything? No prospective trial has selected patients for a particulate therapy on a quantitative receptor or carrier-deposition measurement, so the clinical value of the approach is untested rather than established or refuted.

The order is not arbitrary. The first question gates the others, because until the operative quantity can be measured in patients, the remaining five can be pursued preclinically but not clinically.

## Figures and Tables

**Figure 1:**
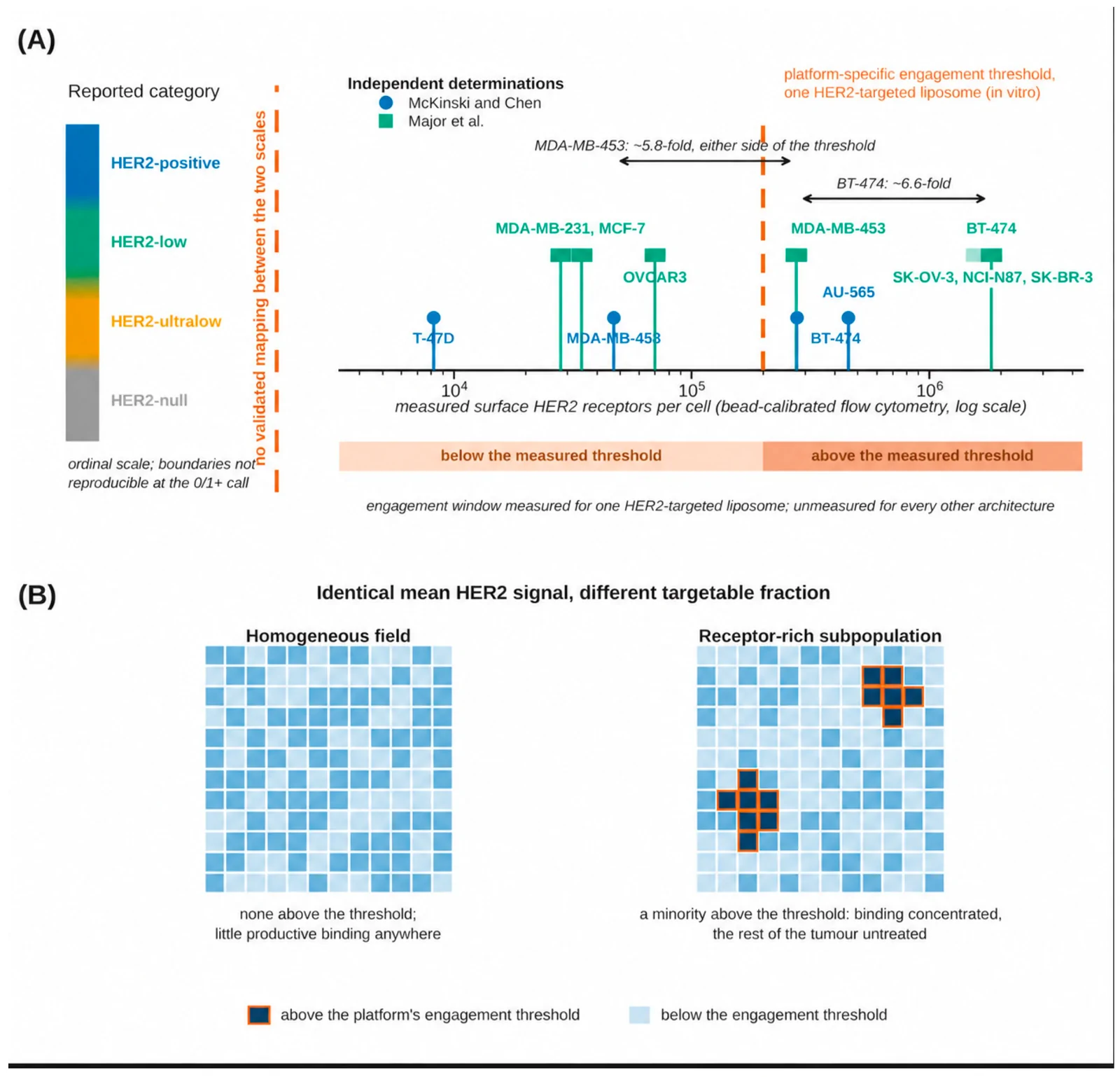
Categorical HER2-reporting and measured receptor abundance are separate scales that cannot be pooled. (**A**) Left panel: the ordinal categories as reported in diagnostic practice, their boundaries drawn graded rather than sharp to signal the approximately 26% reader concordance for the IHC 0 versus 1+ call [22] and the assay-, clone- and specimen-dependent movement of cases across it [27,28]; the gradient is illustrative and carries no scale. The two scales are deliberately not aligned, and no receptor range is assigned to any category, because no validated conversion between them was identified (Section 2.5). The reporting categories are therefore set out on a vertical scale and the measured values on a horizontal one, so that no position on either scale corresponds to a position on the other. Right panel: measured surface HER2-abundance from two independent bead-calibrated flow-cytometric determinations on unpermeabilised cells, keyed by symbol, circles [34] and squares [33]. The two determination-specific series are displayed on a common logarithmic coordinate for visual comparison, but should not be read as a pooled or unified calibration: each is method-specific, and their absolute values cannot be combined. Double-headed arrows mark the two lines measured in both—BT-474, whose values differ by close to sixfold, and MDA-MB-453, which differs by a similar factor and falls on opposite sides of the threshold. No tumour-tissue value is plotted. The dashed line marks the platform-specific engagement threshold of approximately 2 × 10^5^ receptors per cell measured in vitro for one HER2-targeted liposomal doxorubicin [36]; it belongs to that platform alone and is neither a universal nor a clinical threshold. (**B**) Schematic drawing, not a simulation and carrying no data: two tumour fields with the same mean HER2-signal and different spatial distribution, showing that where binding is superselective the fraction of cells above an engagement threshold, rather than the mean, determines how much of a tumour a carrier can engage [40,76].

**Figure 3:**
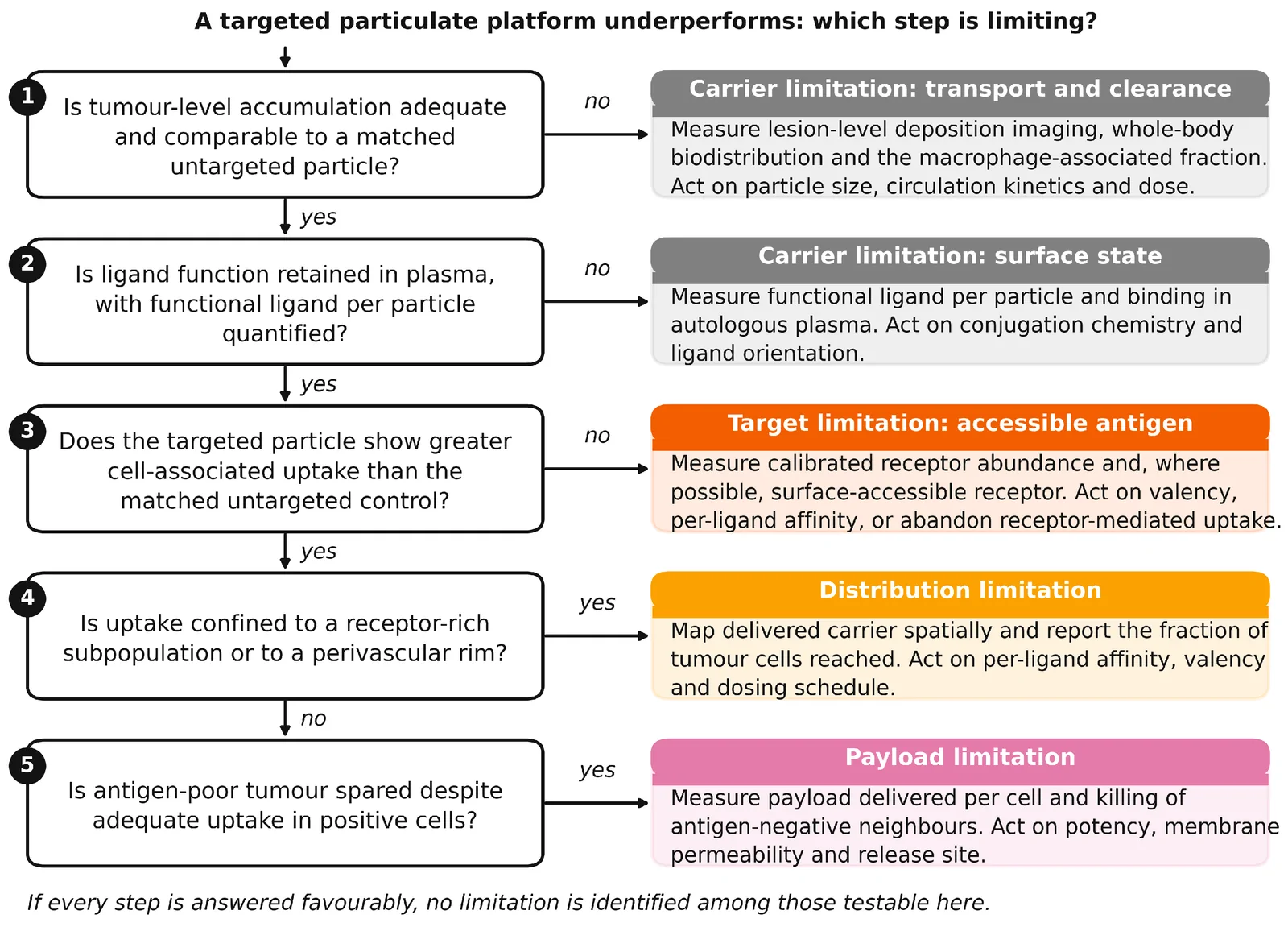
A decision framework for locating the limiting step. An operational sequence for interpreting an underperforming HER2-targeted particulate platform, resolving the five operational limitation classes defined in Section 8.2. Each question specifies an observation that can be made experimentally, and each outcome names the measurement that discriminates it and the design variable it implicates. The two grey outcomes are the carrier classes—transport and clearance limitation, and surface-state limitation—in which too little material arrives or the carrier surface is no longer functional on arrival [39,84,85,108,114]. The vermilion outcome is target limitation, whose signature is loss of the targeted-versus-untargeted difference in cellular uptake at preserved tissue accumulation [36,105]. The amber outcome is distribution limitation, characterised for antibodies and conjugates only [110,111,137]. The pink outcome is payload limitation, for which the quantitative evidence again derives from conjugates [90,91,92]. The framework is diagnostic rather than predictive, ordering existing measurements without assigning weights, and complements the design-variable trade-offs of Section 11 by indicating which parameter is worth changing in a given failure.

**Table 1:** HER2 categories and their implications for particulate targeting. Definitions follow the 2018 ASCO/CAP criteria as reaffirmed in 2023 and the 2026 international consensus reporting recommendations. No receptor-per-cell values are assigned to categories, because no validated conversion was identified.

HER2 Category	Pathological Definition	Biological and Analytical Interpretation	Antigen-Density Limitation	Implication for Particulate Targeting	Key Uncertainty
HER2-positive	IHC 3+, or IHC 2+ with ISH-demonstrated amplification [1,9]	Gene amplification with protein overexpression; the condition the assay was designed and validated to detect	Not usually limiting in the models used to represent it. Bead-calibrated flow cytometry places those lines at 1.42 × 10^6^–2.03 × 10^6^ molecules per cell [33]; a second calibrated determination places BT-474 at 277,096, close to sixfold lower [34]	HER2-overexpressing models used to represent this category exceed the platform-specific engagement threshold measured for one liposomal system [36]. Even so, the one randomised trial of a HER2-targeted nanoparticle identified here was conducted in this category [37] and terminated early without demonstrated benefit, for reasons it cannot resolve [38]	Whether receptor abundance or lesion-level transport limits delivery; deposition varied approximately 35-fold across lesions in a cohort uniformly classified as HER2-positive [39]
HER2-low	IHC 1+, or IHC 2+ without ISH amplification [19]; the component IHC scores follow ASCO/CAP criteria [9]	A therapeutic and regulatory category, not an established biological entity; genomic and transcriptomic analyses show minimal difference from HER2-zero beyond ERBB2 copy number [11,12]	Quantitative assays place most unamplified tumours above the limit of quantification but far below amplified levels, with wide overlap between adjacent IHC categories [30,31]	Not established. No measurement of surface-accessible receptor in tumours of this category was identified, so whether they reach the platform-specific threshold measured for one liposome cannot be stated. Weakly expressing surrogate lines fall below it on one calibrated determination and, for one line, above it on another (Section 3.6) [36,40]	Whether any current particulate architecture engages productively in this range; no in vivo targeted-versus-untargeted comparison across quantified expression was identified
HER2-ultralow	IHC 0 with faint or barely perceptible incomplete membrane staining in >0% to ≤10% of tumour cells [19]	Defined for clinical-trial purposes. The 2023 ASCO/CAP guideline update considered the creation of new HER2 result categories premature at that time, while accepting that distinguishing IHC 0 from 1+ had become clinically relevant [9]; reporting definitions were subsequently provided by the 2026 international consensus [19]	Below the analytical range in which categorical IHC is reproducible; requires a discrimination finer than the 0 versus 1+ call that already shows 26% reader concordance [22]	No calibrated measurement of receptor abundance in this category was identified. The lowest published calibrated tissue assay reported a quantifiable range beginning at 10,375 receptors per cell [34], without validated mapping to the HER2-ultralow category. Productive multivalent engagement is untested rather than excluded, and would be platform-dependent [36,40]	Whether the category identifies a reproducible quantitative interval at all; the supporting clinical subgroup was exploratory with a confidence interval crossing unity [4,6]
HER2-null	IHC 0 with complete absence of membrane staining [19]	Absence of detectable protein by a low-sensitivity assay, not demonstrated absence of receptor; a calibrated assay placed 71% of pathologist-scored IHC 0 cases above its limit of quantification [31]	Not quantified. IHC 0 denotes absence of signal on a low-sensitivity assay, and a high-sensitivity quantitative assay placed 71% of such cases above its limit of quantification [31], so the receptor floor of this category is unknown	Conventional HER2-directed particulate targeting currently has little experimental support in this setting; strategies that install or restore the target, or that do not depend on receptor engagement, are the stated alternatives [41]	Whether responses observed in IHC 0 disease with an ADC reflect residual receptor, assay insensitivity, or receptor-independent mechanisms [42]

Abbreviations: ADC, antibody–drug conjugate; ASCO/CAP, American Society of Clinical Oncology/College of American Pathologists; HER2, human epidermal growth factor receptor 2; IHC, immunohistochemistry; ISH, in situ hybridisation.

**Table 3:** HER2 quantification methods compared by measurand, reported analytical performance and relationship to the surface-accessible receptor pool. Performance figures are those reported in the cited sources; where a source did not report a given characteristic, this is stated rather than estimated. No method is credited with reporting surface-accessible receptors unless it measures receptors available to an extracellular probe on intact cells. The table compares what each method measures and how reproducibly, not the clinical utility of the result, which is established separately (Section 10.1 and Section 10.2).

Method	Measurand and Units	Reported Analytical Performance and Reproducibility	Applicable to Archival Fixed Tissue	Reports Surface-Accessible Receptor	Principal Limitation for Carrier Design	Ref.
Chromogenic immunohistochemistry, ASCO/CAP scoring	Ordinal category (0, 1+, 2+, 3+) from visual read of membrane staining	Reader concordance 26% for 0 versus 1+ against 58% for 2+ versus 3+ [22]; inter-laboratory concordance 68.9% overall (κ = 0.566), falling to 57.6% for IHC 1+ [24]; positive percentage agreement for HER2-0 74.6%, rising to 89.2% after structured training [23]. Limit of detection, limit of quantification, linearity and dynamic range are not defined for an ordinal read and are not reported	Yes	No	Not a measurement of abundance; no validated conversion to receptors per cell; least reproducible at the boundary on which HER2-low depends	[1,9,22,23,24]
Digital image analysis of chromogenic immunohistochemistry	Continuous staining metrics reduced to the same ordinal categories	Agreement against expert consensus raised to weighted κ 0.84 from 0.76, with high specificity for separating ultralow from null tumours in a single-centre series	Yes	No	Improves reproducibility of the existing measurand without calibrating it to receptor number	[155]
Artificial-intelligence-assisted scoring	Ordinal category, algorithmically assisted	Agreement between two pathologists reading 209 slides raised from κ 0.610 to κ 0.713 in HER2-non-amplified cases	Yes	No	Same measurand and same absence of calibration; performance is reader-pair-specific	[24]
Continuous optical-density scoring	Continuous optical density derived from the immunohistochemical stain, arbitrary units	Stratified trastuzumab deruxtecan benefit better than categorical scoring in an exploratory trial analysis; analytical limits not reported in the cited source	Yes	No	Continuous but not calibrated to molecules; exploratory and not prospectively validated	[156]
Quantitative immunofluorescence traceable to mass spectrometry	Continuous protein concentration, attomol/mm^2^	Resolves HER2 in unamplified tumours at 2–20 attomol/mm^2^, with 67% of 364 tumours above the limit of quantification and below amplified levels; a high-sensitivity version placed 71% of pathologist-scored IHC 0 cases above its limit of quantification	Yes	No	Reports fixed-tissue protein per unit area, which includes intracellular receptor and is not the pool available to a carrier	[30,31]
Calibrated quantitative immunohistochemistry with cellular calibrators	Receptors per cell, calibrated against reference cell lines	Reported 10,375–178,649 receptors per cell in tumour tissue, discordant with categorical scoring; two lines measured also by calibrated flow cytometry differed by close to sixfold between the two determinations	Yes	No	The only tissue method reporting molecules per cell, but assay-dependent in absolute value and with a quantifiable range that does not reach the ultralow region	[33,34]
Targeted mass spectrometry on fixed tissue	Absolute protein quantity by selected or multiple reaction monitoring	Absolute quantification against isotope-labelled internal standards; the cited sources report a greater than hundredfold spread of measured HER2 within the IHC 3+/amplified group in gastro-oesophageal tissue. Limits of detection and quantification, linearity and precision are reported in the assay-validation sources and are not reproduced here. Diagnostic discrimination (area under the curve 0.908 for IHC 2+/ISH-negative versus 2+/ISH-positive) is a clinical rather than an analytical characteristic, and is shown here only to mark that distinction	Yes	No	Quantifies total extractable protein in the specimen, with no discrimination of membrane localisation or accessibility	[32,153,154]
Bead-calibrated flow cytometry on unpermeabilised cells	Surface receptors per cell, calibrated against bead standards	Placed HER2-positive lines at 1.42 × 10^6^–2.03 × 10^6^ and weakly expressing or negative lines at 2.81 × 10^4^–2.75 × 10^5^ molecules per cell; a second calibrated determination gave values differing close to sixfold for two shared lines	No	Yes	Reports the relevant pool but requires viable single cells, so it is not applicable to a diagnostic block and has not been reported for HER2-low tumour tissue	[33,34]
Single-molecule localisation microscopy with a labelled extracellular-domain ligand	Surface molecules per µm^2^, cluster radius and clustered fraction	Gave 119, 77 and 4 molecules per µm^2^ for BT-474, SK-BR-3 and MDA-MB-468, cluster radii of 21 and 24 nm and clustered fractions of 49% and 59%, with density against gene copy number fitting at R^2^ = 0.96; applied to freshly excised tissue by touch preparation	No	Yes	The closest available measurement of surface density and its spatial organisation, but not developed into a validated tissue assay and not reported in HER2-low patient tissue	[62]
HER2 positron emission tomography	Tracer uptake per lesion, standardised uptake value	Affibody tracer separated HER2-positive from HER2-negative lesions without overlap; inter- and intra-patient variation in tracer behaviour of up to thirteen- and eightfold; in HER2-low patients uptake did not reach a significant correlation with IHC score (r = 0.41, *p* = 0.24), and maximum standardised uptake value did not reliably separate HER2-low from HER2-positive lesions	Not applicable; in vivo	No	Reports a composite of receptor abundance, tracer delivery, accessibility and retention, which is informative about whole-body heterogeneity and unusable as a receptor measurement	[94,95,96,158,159,160,161,162]
Carrier-deposition imaging	Deposited carrier per lesion, %ID/kg of lesion or relaxivity change	Lesion deposition of a HER2-targeted liposome varied approximately 35-fold (0.52–18.5%ID/kg) and was independent of plasma exposure; an untargeted iron oxide agent varied 8.3-fold across lesions and 1.03- to 4.22-fold within patients	Not applicable; in vivo	No	Measures the delivery endpoint rather than the target, which makes it complementary to, and not a substitute for, receptor quantification	[39,114]
Spatial transcriptomics and single-cell RNA sequencing	ERBB2 transcript abundance, with spatial or single-cell resolution	Resolved significant differences in ERBB2 expression between cancer-cell clusters within individual patients; transcript is an incomplete predictor of protein in general, and transcript-based HER2 measurement showed a high false-negative rate against protein and amplification assays	Yes	No	Measures transcript, not protein and not surface protein; must not be described as a measurement of receptor accessible to a carrier	[76,164,165]
Multiplexed tissue imaging of protein	Tens of proteins per cell with spatial coordinates, relative intensity units	Imaging mass cytometry images tens of proteins at subcellular resolution; cyclic immunofluorescence qualified for breast cancer segmented 201,601 single cells from 567 HER2-positive samples across 189 patients and resolved tumour-cell clusters of heterogeneous HER2 expression	Yes	No	Gives the cell-to-cell distribution of HER2 protein in spatial context, on fixed tissue and without discriminating surface from intracellular receptor	[86,166,167]

Abbreviations: ASCO/CAP, American Society of Clinical Oncology/College of American Pathologists; ERBB2, erb-b2 receptor tyrosine kinase 2 gene; HER2, human epidermal growth factor receptor 2; %ID/kg, percentage of injected dose per kilogram; IHC, immunohistochemistry; ISH, in situ hybridisation. κ denotes Cohen’s or weighted kappa as reported in the source.

**Table 4:** Design variables governing particulate targeting at low antigen density, with trade-offs and the class of evidence supporting each. Evidence classes follow the levels defined in Section 3. Several rows rest on Level D or E sources, and rows drawing on untargeted or generic particulate studies are marked as general particulate evidence outside the HER2-targeting hierarchy.

Design Parameter	Mechanistic Consequence	Potential Benefit at Low Antigen Density	Potential Disadvantage	Evidence Base
Particle diameter	Determines extravasation, interstitial diffusion, membrane wrapping and the number of ligands that can be displayed	Smaller particles may penetrate further and sample more cells distant from vessels	Fewer ligands lowers avidity; less drug per particle; wrapping efficiency falls outside an optimal size range	Level D and E, with general particulate transport evidence outside the HER2-targeting hierarchy [71,72,75,77]
Particle geometry	Modifies contact area, adhesion under shear and non-specific uptake	Unresolved. Elongated shapes raised specific adhesion against endothelial targets and high curvature reduced corona thickness, but neither was tested against tumour antigen density	Manufacturing complexity; shape effects are cell- and flow-dependent	Level D and general particulate evidence; endothelial targeting under flow and cell-free corona measurement, not tumour-antigen targeting [74,79]
Functional ligand density	Sets the number of simultaneous contacts available, and therefore the position of the engagement threshold	An intermediate optimum may raise avidity where receptor is scarce; its position at low receptor density is untested	Most conjugated ligand is not functionally accessible, and excess ligand degraded the target receptor in one system, which targeted CD8 on T cells rather than a tumour antigen	Level B and D; preclinical, several with calibrated ligand counting [52,53,55,56,57,58]
Per-ligand affinity	Governs single-bond lifetime and the sharpness of density discrimination	Lower per-ligand affinity with multivalency may sharpen density discrimination and improve distribution	Predicted benefit diminishes at 10^4^–10^5^ receptors per cell; unvalidated for nanoparticles in vivo	Level C and E; theory, model-membrane and colloid experiments, antibody-format experiments and computational modelling [40,45,48,49,145]
Ligand format and valency	Small scaffolds permit far higher ligand numbers per particle	Higher valency raises total binding in the systems tested, without a proportional gain in selectivity	Higher valency did not translate into proportionally greater selectivity; ligand orientation may matter more than number	Level B; preclinical in vitro [56,83,115]
Ligand spacing and geometry	Determines whether bound ligands can drive receptor clustering and signalling	Defined nanoscale spacing is predicted to raise engagement efficiency without raising ligand number; demonstrated on rigid scaffolds in non-HER2 receptor systems	Achievable mainly on rigid nucleic-acid scaffolds whose pharmacokinetics are unfavourable	Level E; preclinical, defined nucleic-acid scaffolds [59,60,61,147]
Surface chemistry and corona control	Determines whether ligand remains accessible in plasma	Oriented conjugation preserved a substantial fraction of binding in undiluted plasma; high curvature reduced corona thickness, measured by a hybridisation proxy rather than cellular targeting	Corona composition is disease-dependent; untested at low receptor density	Level B and D, with general particulate corona evidence outside the HER2-targeting hierarchy [79,82,83,84,85]
Release mechanism and location	Determines whether therapeutic effect requires internalisation	Extracellular or triggered release would be expected to reduce dependence on receptor-mediated internalisation, the step most sensitive to receptor scarcity	Triggering does not substitute for binding: an ultrasound-triggered immunoliposome gave no benefit in HER2-negative cells	Level B, with one construct outside the HER2-targeting hierarchy [121,122,148]
Tumour transport and carrier deposition	Determines how much carrier reaches the lesion and how uniformly it distributes, before any receptor is engaged	Not determined by tumour-cell antigen density at the arrival and extravasation stages; improved transport would be expected to benefit carrier architectures limited at this step	Not addressable by ligand design. Lies largely upstream of tumour-cell HER2 engagement, although retention and uptake on arrival are not similarly independent of receptor density. Deposition varied by roughly an order of magnitude in each of two human imaging studies, which are separate illustrations rather than comparable measurements (Section 6.1)	General particulate evidence outside the HER2-targeting hierarchy, together with one Level B human dataset [39]; human carrier imaging and quantitative preclinical surveys [39,114,128,131,132]
Payload potency and membrane permeability	Determines whether delivered molecules suffice, and whether antigen-poor neighbours are reached	A potent membrane-permeable payload may reduce dependence on receptor-mediated internalisation and broaden activity across heterogeneous expression; demonstrated for conjugates only	The effect is local and diffusion-limited; permeability raises normal-tissue exposure; unquantified for particulate carriers	Level C; conjugate-based preclinical evidence [90,91,92,99]

Abbreviations: CD8, cluster of differentiation 8; HER2, human epidermal growth factor receptor 2. Evidence levels A–E are defined in Section 3.

## Data Availability

No new data were created or analysed in this study. Data sharing is not applicable to this review.

## Abbreviations

ADC, antibody–drug conjugate; ASCO, American Society of Clinical Oncology; CAP, College of American Pathologists; CI, confidence interval; DARPin, designed ankyrin repeat protein; HER2, human epidermal growth factor receptor 2; HR, hazard ratio; %ID/g, percentage of injected dose per gram; %ID/kg, percentage of injected dose per kilogram; IHC, immunohistochemistry; ISH, in situ hybridisation; LNP, lipid nanoparticle; MMAE, monomethyl auristatin E; MTD, maximum tolerated dose; ORR, objective response rate; PAMAM, poly(amidoamine); PEG, poly(ethylene glycol); PET, positron emission tomography; PFS, progression-free survival; SANRA, Scale for the Assessment of Narrative Review Articles; scFv, single-chain variable fragment; T-DM1, ado-trastuzumab emtansine; T-DXd, trastuzumab deruxtecan; VHH, single-domain antibody variable region.
